# Hepatitis B virus e antigen induces atypical metabolism and differentially regulates programmed cell deaths of macrophages

**DOI:** 10.1371/journal.ppat.1012079

**Published:** 2024-03-11

**Authors:** Yumei Li, Christine Wu, Jiyoung Lee, Qiqi Ning, Juhyeon Lim, Hyungjin Eoh, Sean Wang, Benjamin P. Hurrell, Omid Akbari, Jing-hsiung James Ou

**Affiliations:** 1 Department of Molecular Microbiology and Immunology, Keck School of Medicine, University of Southern California, Los Angeles, California, United States of America; 2 Michael Amini Transfusion Medicine Center, City of Hope, Duarte, California, United States of America; Pennsylvania State University College of Medicine: Penn State College of Medicine, UNITED STATES

## Abstract

Macrophages can undergo M1-like proinflammatory polarization with low oxidative phosphorylation (OXPHOS) and high glycolytic activities or M2-like anti-inflammatory polarization with the opposite metabolic activities. Here we show that M1-like macrophages induced by hepatitis B virus (HBV) display high OXPHOS and low glycolytic activities. This atypical metabolism induced by HBV attenuates the antiviral response of M1-like macrophages and is mediated by HBV e antigen (HBeAg), which induces death receptor 5 (DR5) via toll-like receptor 4 (TLR4) to induce death-associated protein 3 (DAP3). DAP3 then induces the expression of mitochondrial genes to promote OXPHOS. HBeAg also enhances the expression of glutaminases and increases the level of glutamate, which is converted to α-ketoglutarate, an important metabolic intermediate of the tricarboxylic acid cycle, to promote OXPHOS. The induction of DR5 by HBeAg leads to apoptosis of M1-like and M2-like macrophages, although HBeAg also induces pyroptosis of the former. These findings reveal novel activities of HBeAg, which can reprogram mitochondrial metabolism and trigger different programmed cell death responses of macrophages depending on their phenotypes to promote HBV persistence.

## Author summary

Hepatitis B virus (HBV) causes chronic infection in approximately 300 million people in the world, resulting in nearly 1 million deaths per year. How HBV establishes chronic infection in patients is an interesting question. Hepatic macrophages play important role in HBV clearance and persistence. They can be induced by external stimuli to display different metabolic activities. Here we demonstrates that HBV can reprogram the metabolism of macrophages to induce oxidative phosphorylation to attenuate their anti-HBV activities and in the meantime differentially regulate programmed cell deaths of macrophages based on their phenotypes. These results provide important information for understanding the mechanism of HBV persistence and pathogenesis and offer a new avenue for the development of novel treatments for HBV patients.

## Introduction

Macrophages are phenotypically plastic and can undergo the M1 proinflammatory polarization or the M2 anti-inflammatory polarization with some variations depending on the environmental cues [1,2]. M1 macrophages are characterized by their ability to produce reactive oxygen species (ROS) and proinflammatory cytokines such as tumor necrosis factor-α (TNF-α) and IL-1β. In contrast, M2 macrophages are characterized by their expression of anti-inflammatory genes such as CD163, which is a member of the scavenger receptor superfamily, and IL-10, which is an anti-inflammatory cytokine. These two types of macrophages also have distinct metabolic profiles, with M1 macrophages displaying a low oxidative phosphorylation (OXPHOS) activity and a high glycolytic activity, and M2 macrophages displaying a high OXPHOS activity and a low glycolytic activity [3–5]. These distinct metabolic activities are important for the functionalities of M1 and M2 macrophages, although recent studies indicated that this dichotomous separation of M1 and M2 metabolic phenotypes might be oversimplified [5].

Hepatitis B virus (HBV) is an important human pathogen. This liver-tropic virus chronically infects approximately 300 million people in the world and can cause severe liver diseases including cirrhosis and hepatocellular carcinoma (HCC). HBV can induce Kupffer cells, the resident macrophages of the liver, to undergo either the M1-like or the M2-like polarization, resulting in either viral clearance or persistence [6]. HBV is a small DNA virus with a genome size of approximately 3.2 kb. Its genome contains four genes named S, C, X, and P genes. The S gene codes for viral envelope protein known as the surface antigen (HBsAg). The C gene codes for the core protein, which forms the viral core particle that displays a conformational epitope known as the core antigen (HBcAg), as well as a related protein named the e antigen (HBeAg), which is secreted from HBV-infected hepatocytes [7]. The X gene codes for a regulatory protein named the X protein (HBx), and the P gene codes for the viral DNA polymerase (for a review, see [8]).

Recently, we discovered that HBV could promote OXPHOS in M1-like macrophages to attenuate the production of the pro-inflammatory cytokine IL-1β [9]. In this report, we further studied how HBV might impact the metabolism of macrophages. We found that HBV could enhance OXPHOS in macrophages and this enhancement was dependent on HBeAg. Our further studies indicated that the effect of HBeAg on macrophages was mediated by toll-like receptor 4 (TLR4), which induced the expression of death receptor 5 (DR5) and death-associated protein 3 (DAP3) to enhance the expression of mitochondrial enzymes and glutaminases (GLS) to promote OXPHOS. The ability of HBeAg to induce DAP3 was unique, as lipopolysaccharides (LPS), which also activated TLR4, was not able to induce DAP3 and promote OXPHOS. We also found that HBeAg triggered pyroptosis and apoptosis of M1-like macrophages and primarily apoptosis of M2-like macrophages. These findings provided mechanistic details for understanding how HBV metabolically reprogrammed macrophages to attenuate their antiviral activities and demonstrated that HBV could induce programmed cell deaths of macrophages in a phenotype-dependent manner.

## Results

### HBV induces atypical metabolism of M1-like macrophages

**Fig 1:**
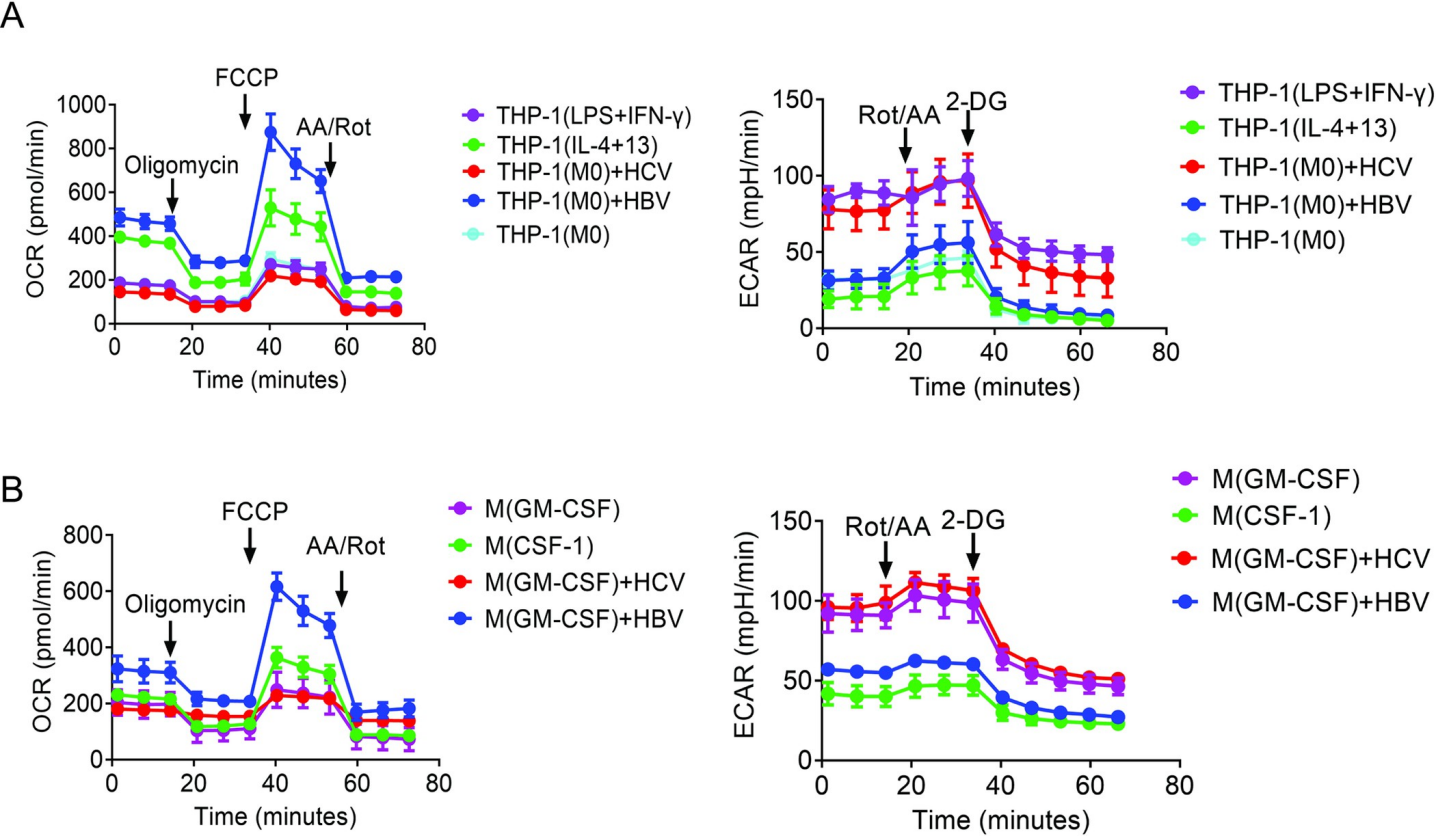
Macrophages treated with HBV displayed high OXPHOS and low glycolytic activities. (A) Analysis of OCR (left panel) and ECAR (right panel) of THP-1-derived macrophages (THP-1(M0)) that were treated with LPS+ IFN-γ (THP-1(LPS+IFN-γ)), IL-4+IL-13 (THP-1(IL-4+13)), HCV (THP-1(M0)+HCV) or HBV (THP-1(M0)+HBV). THP-1(M0) that was not treated was used as the negative control. (B) Analysis of OCR (left panel) and ECAR (right panel) of human M1-like macrophages (M(GM-CSF)), M2-like macrophages (M(CSF-1)), and M(GM-CSF) treated with HCV (M(GM-CSF)+HCV) or HBV (M(GM-CSF)+HBV).

To determine how HBV might affect the metabolism of macrophages, we treated THP-1 cells, a human monocytic cell line, with phorbol-12-myristate-13-acetate (PMA) to induce their differentiation into CD14^+^CD11b^+^ macrophage (i.e., M0 macrophages). These M0 THP-1 macrophages were then treated either with LPS and interferon-γ (IFN-γ) for the induction of M1-like polarization or with IL-4 and IL-13 for the induction of M2-like polarization as previously described [9]. The M1-like and M2-like phenotypes of THP-1 macrophages were confirmed by the analysis of M1 markers IL-1β, TNF-α and inducible nitric oxide synthase (iNOS) and M2 markers CD163, IL-10 and mannose receptor C-type 1 (MRC-1) (S1A Fig). These M1-like and M2-like macrophages were used as the controls. In addition, we also treated M0 THP-1 macrophages with the incubation media harvested from Huh7 hepatoma cells that had been transfected with the HBV genomic DNA or infected with hepatitis C virus (HCV), which is also a hepatotropic virus for comparison. THP-1 macrophages treated with HBV or HCV displayed M1-like phenotypes, as they expressed increased levels of IL-1β, TNF-α and iNOS and reduced levels of CD163, IL-10 and MRC-1 (S1A Fig). These THP-1 macrophages were then subjected to Seahorse analysis for the determination of their oxygen consumption rate (OCR) and the extracellular acidification rate (ECAR), which measured OXPHOS and glycolytic activities, respectively [10]. As shown in Fig 1A, M1-like THP-1(LPS+IFN-γ) macrophages had a low OCR and a high ECAR and M2-like THP-1(IL-4+13) macrophages had the opposite metabolic activities, in agreement with the previous reports (for a review, see [11]). While THP-1 macrophages treated with HCV had a low OCR and a high ECAR, as would be expected for M1-like macrophages, THP-1 macrophages treated with HBV had a high OCR and a low ECAR (Fig 1A), indicative of high OXPHOS and low glycolytic activities. These results indicated that, while both HBV and HCV could induce the M1-like polarization of macrophages, only HBV could induce the atypical M1 metabolism with high OXPHOS and low glycolytic activities.

To ensure that the effects of HBV and HCV were not specific to THP-1 macrophages, we also isolated human CD14^+^CD16^-^ classical monocytes from the blood of healthy donors and treated them with GM-CSF (i.e., M(GM-CSF)) or CSF-1 (i.e., M(CSF-1)) as previously described [9]. M(GM-CSF) and M(CSF-1) displayed M1-like and M2-like phenotypes, respectively, which were confirmed by the analysis of M1 and M2 markers (S1B Fig). Interestingly, although HCV could further increase the expression levels of IL-1β, TNF-α and iNOS without increasing the expression levels of CD163, IL-10 and MRC-1 to enhance the M1-like phenotype of M(GM-CSF) macrophages, HBV reduced the expression levels of IL-1β, TNF-α and iNOS and simultaneously increased the expression levels of CD163, IL-10 and MRC-1, resulting in the attenuation of the M1-like phenotype of M(GM-CSF) macrophages (S1B Fig). These human monocyte-derived macrophages (MDMs) were then subjected to Seahorse analysis. As shown in Fig 1B, the same as THP-1 macrophages, the M1-like M(GM-CSF) macrophages had a low OCR and a high ECAR whereas the M2-like M(CSF-1) macrophages had an elevated OCR and low ECAR. M(GM-CSF) treated with HCV had a metabolic profile similar to that of M(GM-CSF). In contrast, M(GM-CSF) treated with HBV had a much higher OCR and a lower ECAR than M(GM-CSF). These results indicated that HBV, but not HCV, could metabolically reprogram not only THP-1 macrophages but also M1-like human MDMs to promote OXPHOS and reduce glycolysis. The quantitative analysis of various metabolic parameters of OCR and ECAR results are shown in S1C–S1F Fig.

### HBeAg mediates the effect of HBV to induce OXPHOS in macrophages both *in vitro* and *in vivo*

**Fig 2:**
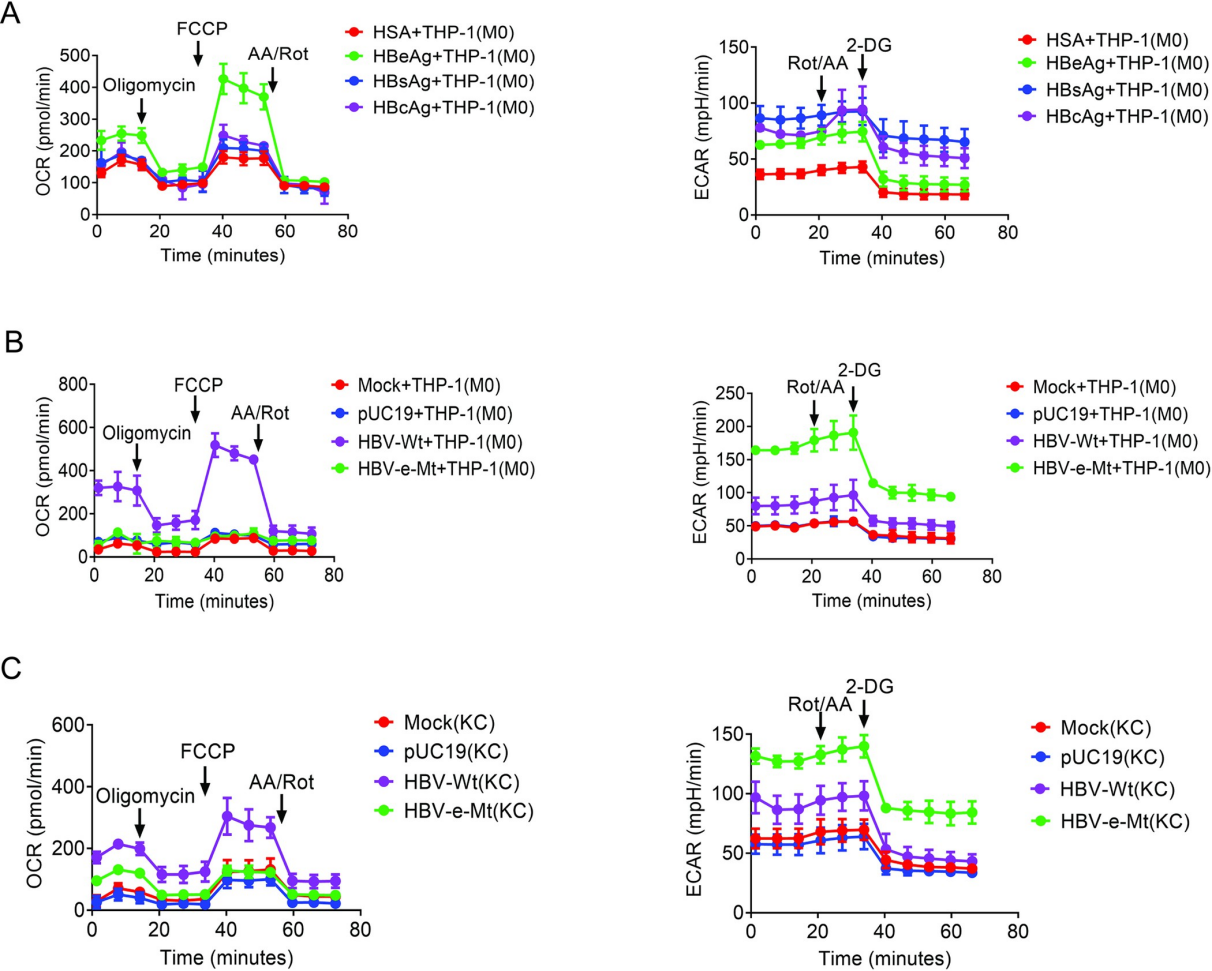
HBeAg induces OXPHOS and suppresses glycolysis induced by HBV both *in vitro* and *in vivo*. (A) Analysis of OCR (left panel) and ECAR (right panel) of THP-1 macrophages that were treated with recombinant 1 μg/mL HSA, HBeAg, HBsAg or HBcAg for 48 hours. (B) Analysis of OCR (left panel) and ECAR (right panel) of THP-1 macrophages with the incubation medium harvested from Huh7 cells transfected with pUC19, the 1.3mer wild-type HBV genomic DNA or the 1.3mer HBV genomic DNA that was incapable of expressing only HBeAg (HBV-e-Mt). (C) Analysis of OCR (left panel) and *ECAR* (right panel) of Kupffer cells (KC) isolated from mice without (Mock) or with the hydrodynamic injection of pUC19, 1.3mer wild-type HBV DNA or 1.3mer HBeAg-null HBV DNA.

HBeAg, HBsAg and HBcAg are three different HBV antigens that are released from HBV-infected hepatocytes. To determine whether the effect of HBV on macrophages was mediated by any of these three different antigens, we treated THP-1 M0 macrophages with recombinant HBeAg, HBsAg, HBcAg or the control recombinant human serum albumin (HSA) followed by Seahorse analysis. As shown in Fig 2A, comparing with HSA, HBsAg and HBcAg had little effect on the OCR of THP-1 macrophages. However, HBeAg was able to significantly increase it. When the ECAR was analyzed, macrophages treated by all three HBV antigens had a higher ECAR than those treated with HSA, with HBeAg having the least effect among the three HBV antigens. The quantitative analysis of the metabolic parameters of OCR and ECAR results are shown in S2A and S2B Fig. These results indicated that HBeAg, but not HBsAg or HBcAg, could induce the OXPHOS in THP-1 macrophages, and, as shown in S2C Fig, this induction was dose-dependent with higher doses of HBeAg inducing higher levels of OXHPOS.

To determine whether HBeAg was essential for HBV to induce the OXPHOS of macrophages, we treated THP-1 M0 macrophages with the incubation media harvested from Huh7 cells that had been transfected with the control vector pUC19, the 1.3mer wild-type HBV genomic DNA, or the 1.3mer HBV mutant DNA that was incapable of expressing only HBeAg. In agreement with the results shown in Fig 1, the wild-type HBV was able to significantly increase the OCR with only a marginal effect on the ECAR (Figs 2B, S2D and S2E). In contrast, the HBV mutant that was incapable of expressing HBeAg could not increase the OCR in macrophages and instead significantly increased the ECAR. Similar results were obtained *in vivo* using mice as a model. We isolated Kupffer cells (i.e., hepatic macrophages) from mice four days after hydrodynamic injection with the pUC19 vector, the 1.3mer wild-type HBV genomic DNA or the 1.3mer HBeAg-null HBV genomic DNA for Seahorse assay. At this timepoint, the serum HBV titers in mice injected with the wild-type DNA and the HBeAg-null HBV DNA were similar at about 10^7^ genome-equivalent per ml [6]. As shown in Figs 2C, S2F and S2G, wild-type HBV, but not the HBeAg-null HBV, was able to increase the OCR of Kupffer cells. These results together indicated that HBeAg was essential for HBV to induce the OXPHOS in macrophages both *in vitro* and *in vivo*. In addition, as the HBeAg-null mutant increased the ECAR to a much higher level than that induced by the wild-type HBV in both THP-1 macrophages and mouse Kupffer cells, HBeAg apparently could also suppress glycolysis induced by other HBV antigens.

### HBeAg induces DAP3 to promote the OXPHOS in macrophages

**Fig 3:**
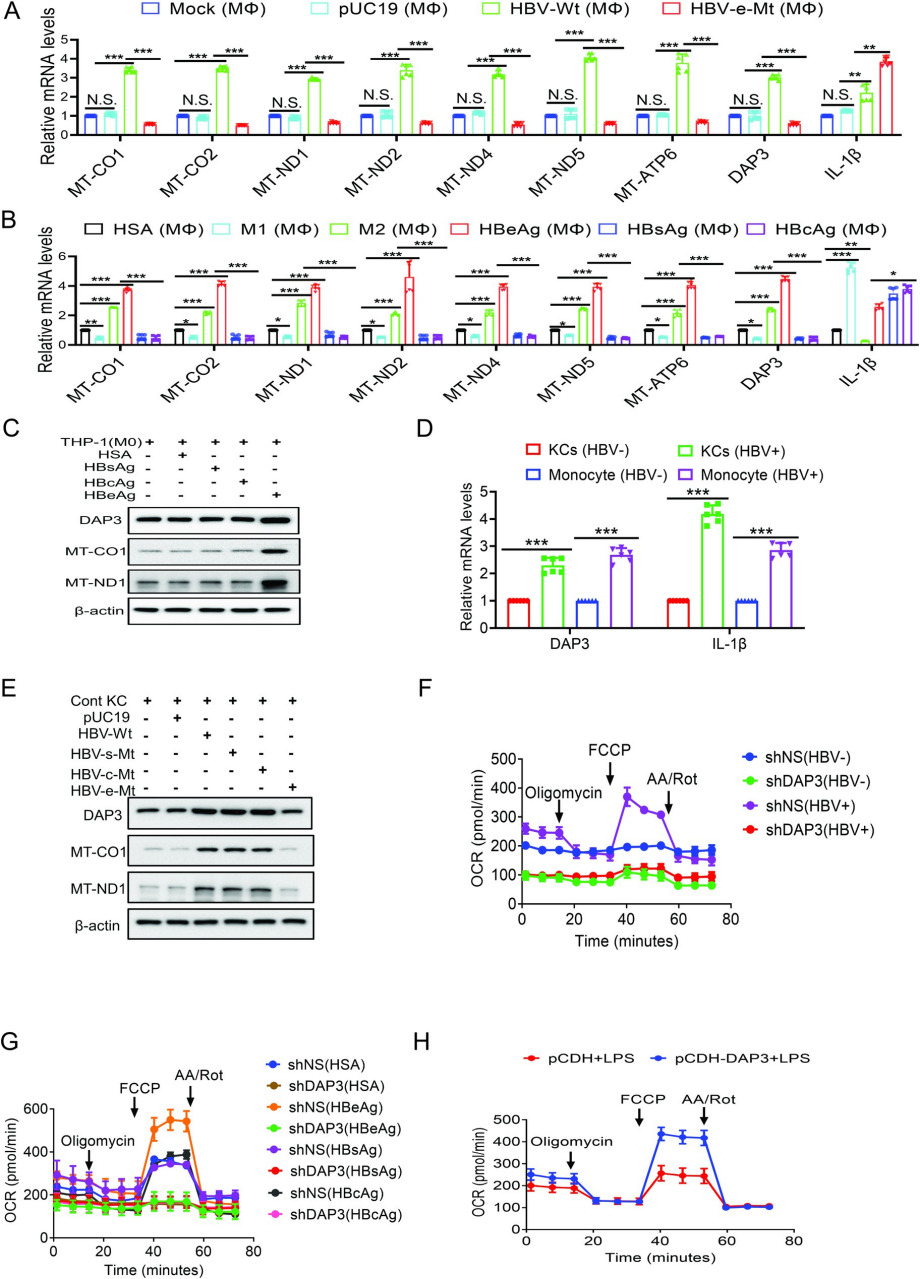
HBeAg induces DAP3 and mitochondrial proteins to promote OXPHOS of macrophage both *in vitro* and *in vivo*. (A) THP-1 M0 macrophages treated with the incubation medium of Huh7 cells that had been transfected with pUC19, the 1.3mer wild-type HBV genomic DNA or the 1.3mer HBeAg-null HBV genomic DNA were analyzed by RT-qPCR for the RNA levels of mitochondrial proteins, DAP3 and IL-1β. (B) THP-1 M0 macrophages treated with HSA, LPS+IFN-γ (i.e., M1 macrophages), IL4+IL13 (i.e., M2 macrophages), 1 μg/mL HBeAg, HBsAg or HBcAg for 48 hours were analyzed by RT-qPCR for the expression of mitochondrial genes, DAP3 and IL-1β. (C) THP-1 macrophages treated with 1 μg/mL HSA, HBsAg, HBcAg or HBeAg for 48 hours were lysed for immunoblot analysis. β-actin served as a loading control. (D) Human MDMs with (HBV+) and without (HBV-) treatment and Kupffer cells that were isolated from mice that had been injected with pUC19 (HBV-) or the 1.3mer HBV genomic DNA (HBV+) were analyzed for DAP3 and IL-1β RNAs by RT-qPCR. (E) Kupffer cells isolated from mice three days after the injection of pUC19, 1.3mer wild-type HBV genomic DNA or the HBV genomic DNA mutant that was incapable of expressing HBsAg, HBcAg or HBeAg were lysed for immuoblot analysis. (F) THP-1 macrophages were transduced with a lentiviral vector that expressed a nonspecific shRNA (shNS) or the DAP3 shRNA (shDAP3) and then treated with the incubation medium of Huh7 cells that were transfected with pUC19 (HBV-) or the 1.3mer HBV genomic DNA (HBV+). The OCR of THP-1 macrophages was then measured by Seahorse assay. (G) THP-1 macrophages with and without DAP3 silencing as in (F) were treated with 1 μg/mL HSA, HBsAg, HBcAg or HBeAg for 48 hours and then subjected to Seahorse analysis of OCR. (H) THP-1 macrophages were transduced with a control lentiviral vector or a lentiviral vector that expressed DAP3, stimulated with 100 ng/mL LPS for 24 hours and then subjected to Seahorse analysis of OCR. In (A), (B) and (D), N.S., statistically not significant; *, *p*<0.05; **, *p*<0.01; ***, *p*<0.001.

By conducting the RNA-seq analysis, we had previously found that HBV could induce the expression of mitochondrial genes that are involved in the OXPHOS of macrophages [9]. To determine whether this effect of HBV on mitochondrial genes was dependent on HBeAg, we analyzed the RNA levels of these mitochondrial genes by RT-qPCR. As shown in Fig 3A, in agreement with our previous results, THP-1 macrophages treated with the incubation medium of Huh7 cells that had been transfected with the wild-type HBV genomic DNA had increased expression levels of mitochondrial genes including MT-CO1, MT-CO2, MT-ND1, MT-ND2, MT-ND4, MT-ND5, and MT-ATP6. These mitochondrial genes encode enzymes that are required for the OXPHOS. HBV also increased the expression level of death-associated protein 3 (DAP3), a protein encoded by the chromosomal DNA in the nucleus. DAP3 is involved in extrinsic apoptosis of cells [12]. However, it is also known as S29, a protein of the 28S ribosomal subunit of mitochondria that regulates the translation of mitochondrial proteins [13]. The increased expression of mitochondrial genes and DAP3 was abolished and even further reduced when THP-1 macrophages were treated with the incubation medium of Huh7 cells that had been transfected with the HBeAg-negative HBV genomic DNA. In contrast, the loss of HBeAg did not abolish but rather, further increased the expression of IL-1β, a pro-inflammatory cytokine that was induced by HBV (Fig 3A). These results indicated that HBeAg was critical for HBV to induce the expression of mitochondrial genes and DAP3, and that HBeAg could attenuate the expression of IL-1β induced by other HBV gene products (see below).

To further determine whether HBeAg by itself was sufficient to induce the expression of mitochondrial genes and DAP3, we treated THP-1 M0 macrophages with HSA, HBeAg, HBsAg or HBcAg and then analyzed the expression of these mitochondrial genes and DAP3 as well as IL-1β in cells. We also analyzed the expression of these genes in M1-like and M2-like THP-1 macrophages. As shown in Fig 3B, comparing with THP-1 macrophages that were treated with the control HSA recombinant protein, M1-like THP-1 macrophages had a reduced expression level of mitochondrial genes and DAP3 whereas M2-like macrophages had an increased expression level of these genes. These results were consistent with the observations that M1 macrophages had low OXPHOS activities and M2 macrophages had high OXPHOS activities [4,5]. The expression levels of mitochondrial genes and DAP3 in THP-1 macrophages were significantly increased by HBeAg, in agreement with the induction of OXPHOS in THP-1 macrophage by HBeAg (Fig 2A). In contrast, they were reduced by HBsAg and HBcAg (Fig 3B), similar to that observed in M1-like macrophages. The expression level of IL-1β was increased in M1-like macrophages and by all three HBV antigens analyzed, with HBeAg having the least effect. To confirm the RNA results, we also selectively analyzed the protein levels of DAP3 and two mitochondrial enzymes MT-CO1 and MT-ND1. As shown in Fig 3C, in agreement with the RNA results, HBeAg, but not HBsAg or HBcAg, increased the protein levels of DAP3, MT-CO1 and MT-ND1 in THP-1 macrophages.

To ensure that the effect of HBV on DAP3 and mitochondrial genes was not limited to THP-1 macrophages, we also analyzed human MDMs with and without the treatment of HBV and Kupffer cells that were isolated from mice that had been injected with pUC19 or the HBV genomic DNA. As shown in Fig 3D, HBV also increased the expression levels of DAP3 and IL-1β in human MDMs and mouse Kupffer cells. We also conducted the immunoblot analysis on Kupffer cells that were isolated from mice that had been injected with the 1.3mer wild-type HBV genomic DNA or HBV genomic DNA with mutations that abolished the expression of HBsAg, HBcAg or HBeAg. As shown in Fig 3E, although HBV wild-type, HBsAg mutant and HBcAg mutant could induce the expression of DAP3, MT-CO1 and MT-ND1, HBeAg mutant could not. These results together indicated that HBeAg was essential and sufficient for HBV to induce the expression of mitochondrial genes and DAP3 and that the effect of HBeAg on the expression of these genes was not specific to THP-1 macrophages and could also be detected in human MDMs *ex vivo* and in mouse Kupffer cells *in vivo*.

To determine whether DAP3 and mitochondrial genes induced by HBV is indeed important for the OXPHOS in macrophages, we performed the RNA silencing experiment. We focused our attention on DAP3, as it regulates the translation of mitochondrial proteins, by silencing its expression with shRNA (S3A and S3B Fig). Interestingly, the silencing of DAP3 reduced the RNA levels of mitochondrial genes in THP-1 macrophages with or without the HBV stimulation (S3C Fig). This result indicated that, in addition to participating in the translation of mitochondrial proteins, DAP3 might also regulate the transcription and/or the RNA stability of mitochondrial genes. The silencing of DAP3 also increased the expression of IL-1β, particularly in THP-1 macrophages that were stimulated with HBV, indicating that DAP3 was a negative regulator for IL-1β expression. The Seahorse analysis indicated that the silencing of DAP3 reduced the OCR of THP-1 macrophages with a more pronounced effect on THP-1 macrophages that were treated with HBV (Figs 3F and S3D). Similarly, DAP3 silencing also reduced the OCR of THP-1 macrophages that were treated with HSA, HBsAg, HBcAg or HBeAg to the basal level (Figs 3G and S3E). Interestingly, the silencing of DAP3 also increased the ECAR of THP-1 macrophages that were treated with HBeAg (S3F Fig), indicating a negative effect of DAP3 on glycolysis. In contrast, the over-expression of DAP3 significantly increased the OCR of THP-1 macrophages that were treated with LPS (Figs 3H and S3G). These results together demonstrated an important role of DAP3 in the OXPHOS and were consistent with the notion that HBeAg induced DAP3 to promote OXPHOS in macrophages.

### HBV increases the level of glutamate to promote OXPHOS in macrophages

**Fig 4:**
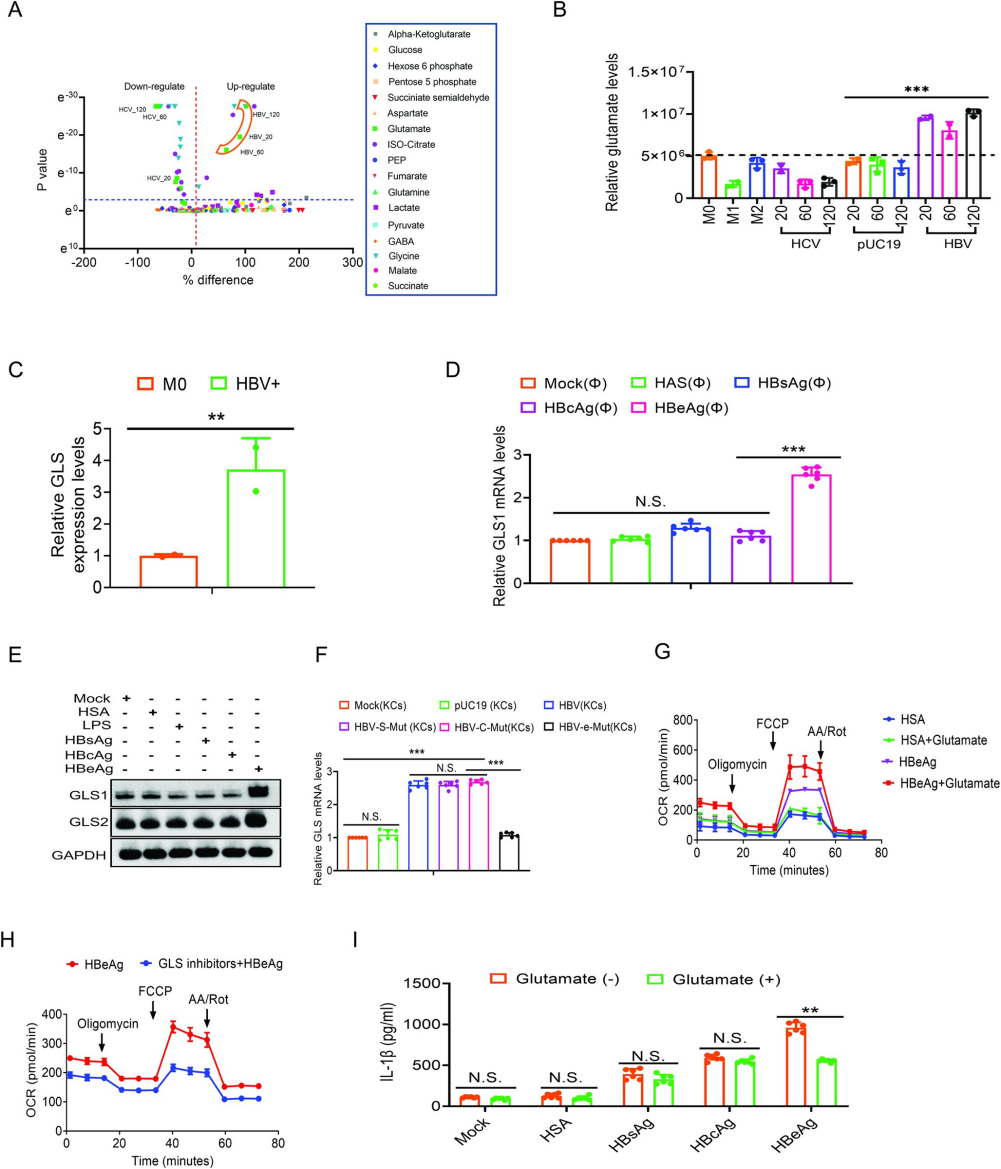
HBeAg induces GLS and increases glutamate to promote OXPHOS. (A) THP-1 M0 macrophages with various treatments were subjected to metabolomic analysis. The volcano plot displays changes of metabolites induced by the treatment of LPS+IFN-γ, IL-4+IL-13, pUC19, HBV or HCV. The boxed area denotes glutamate that was increased by different doses of HBV. In contrast, HCV reduced glutamate levels. (B) The levels of glutamate in THP-1 macrophages with different treatments as shown in (A) were compared. In both (A) and (B), 20, 60 and 120 indicated the volume (in μL) of the incubation media used to treat THP-1 macrophages. M1, THP-1 macrophages treated with LPS+IFN-γ; M2, THP-1 macrophages treated with IL-4+IL-13. (C) The GLS1 RNA of THP-1 macrophages with or without the treatment of HBV were quantified by RT-qPCR. (D) The GLS1 RNA in THP-1 macrophages without or with the treatment of 1 μg/mL HSA, HBsAg, HBcAg or HBeAg were quantified by RT-qPCR. (E) THP-1 macrophages treated with 1 μg/mL HSA, LPS, HBsAg, HBcAg or HBeAg were lysed for immunoblot analysis of GLS1 and GLS2. GAPDH served as the loading control. (F) Kupffer cells isolated from mice that had been injected with pUC19 or the 1.3mer HBV genomic DNA without or with mutations that abolished the expression of HBsAg, HBcAg or HBeAg were analyzed for the levels of GLS1 RNA by RT-qPCR. Mice not injected with DNA (mock) was used as the control. (G) THP-1 macrophages (MΦ) with or without the treatment of 20 mM glutamate HSA or HBeAg were subjected to Seahorse analysis for their OCRs. (H) THP-1 macrophages were treated with HBeAg in the presence or absence of GLS inhibitors Telaglenastat (1 μM) and compound 968 (5 μM) and then subjected to Seahorse analysis for their OCR. (I) THP-1 macrophages without (-) or with (+) the treatment of 20 mM glutamate were not treated or treated with 1 μg/mL HSA, HBsAg, HBcAg or HBeAg for 48 hours. The incubation media were then harvested for the measurement of IL-β by ELISA. In (B-D), (F) and (I), N.S., not significant; **, *p*<0.01; ***, *p*<0.001.

To further understand how HBV might have affected the metabolism of THP-1 macrophages, we conducted the metabolomic analysis on M1-like and M2-like THP-1 macrophages and THP-1 macrophages that were treated with either HBV or HCV. As shown in the volcano plot in Fig 4A, glutamate was a prominent metabolite that was induced by HBV. In contrast, it was reduced by HCV. A detailed analysis of the glutamate levels in THP-1 macrophages with different treatments is shown in Fig 4B. As shown in the chart, the M1-like polarization reduced the glutamate level whereas the M2-like polarization had only a marginal effect. Similar to M1-like macrophages, HCV also reduced the glutamate levels in more or less a dose-dependent manner. In contrast, HBV significantly increased the glutamate level in THP-1 macrophages even at a low dose. To understand how HBV might have increased the glutamate level, we examined the RNA-seq data that we had previously generated from macrophages for genes that might be induced by HBV and are involved in glutamate synthesis [9]. Interestingly, as shown in Fig 4C, HBV increased the RNA level of glutaminase-1 (GLS1), which mediates glutaminolysis to convert glutamine to glutamate, approximately 3-fold in THP-1 macrophages. The treatment of THP-1 macrophages with HBeAg, but not HSA, HBsAg or HBcAg, also increased the GLS1 RNA level two to three-fold (Fig 4D). Our immunoblot analysis confirmed that HBeAg, but not LPS, HBsAg or HBcAg, could indeed increase the GLS1 protein level (Fig 4E). A slight increase of another glutaminase GLS2 by HBeAg was also observed. The ability of HBV to enhance the expression of GLS1 in an HBeAg-dependent manner *in vivo* was also observed when mouse Kupffer cells isolated from mice that had been injected with the HBV genomic DNA were analyzed (Fig 4F).

Glutamate can be converted to α-ketoglutarate (αKG), a metabolic intermediate of the tricarboxylic (TCA) cycle [14]. To determine whether the increase of glutamate could promote the OXPHOS, we added glutamate into the incubation medium of THP-1 macrophages followed by the treatment of HSA or HBeAg. As shown in Figs 4G and S4A, although the supplementation of glutamate had no effect on the OCR of HSA-treated macrophages, it increased the OCR of HBeAg-treated THP-1 macrophages. Conversely, the treatment of HBeAg-treated THP-1 macrophages with Telaglenastat and compound 968, which are GLS inhibitors, reduced the OCR (Figs 4H and S4B). These results suggested an important role of glutaminolysis in HBeAg-induced OXHPOS. To further test this possibility, we conducted ^13^C-tracing experiment using uniformly ^13^C-labeled [U-^13^C] glutamine. As shown in S4C Fig, ^13^C_5_-glutamine was converted to ^13^C_5_-glutamate, ^13^C_5_-αKG and, after αKG decarboxylation, ^13^C_4_ isotopologues of other metabolic intermediates of the TCA cycle in HBeAg-treated THP-1 macrophages. However, very little αKG derived from ^13^C_5_-glutamine was detected in mock-treated cells. These results supported the model that HBeAg increased the level of glutamate and enhanced its conversion to αKG to promote the TCA cycle and the OXPHOS.

Finally, we analyzed the effect of glutamate on the production of IL-1β. As shown in Fig 4I, although the addition of glutamate had no effect on the release of IL-1β from THP-1 macrophages treated with HBsAg or HBcAg, it reduced the release of IL-1β from THP-1 macrophages treated with HBeAg. This result indicated that glutamate, which promoted the OXPHOS in macrophages treated with HBeAg, also suppressed the production of IL-1β.

### HBeAg induces apoptosis of macrophages in a phenotype-dependent manner

**Fig 5:**
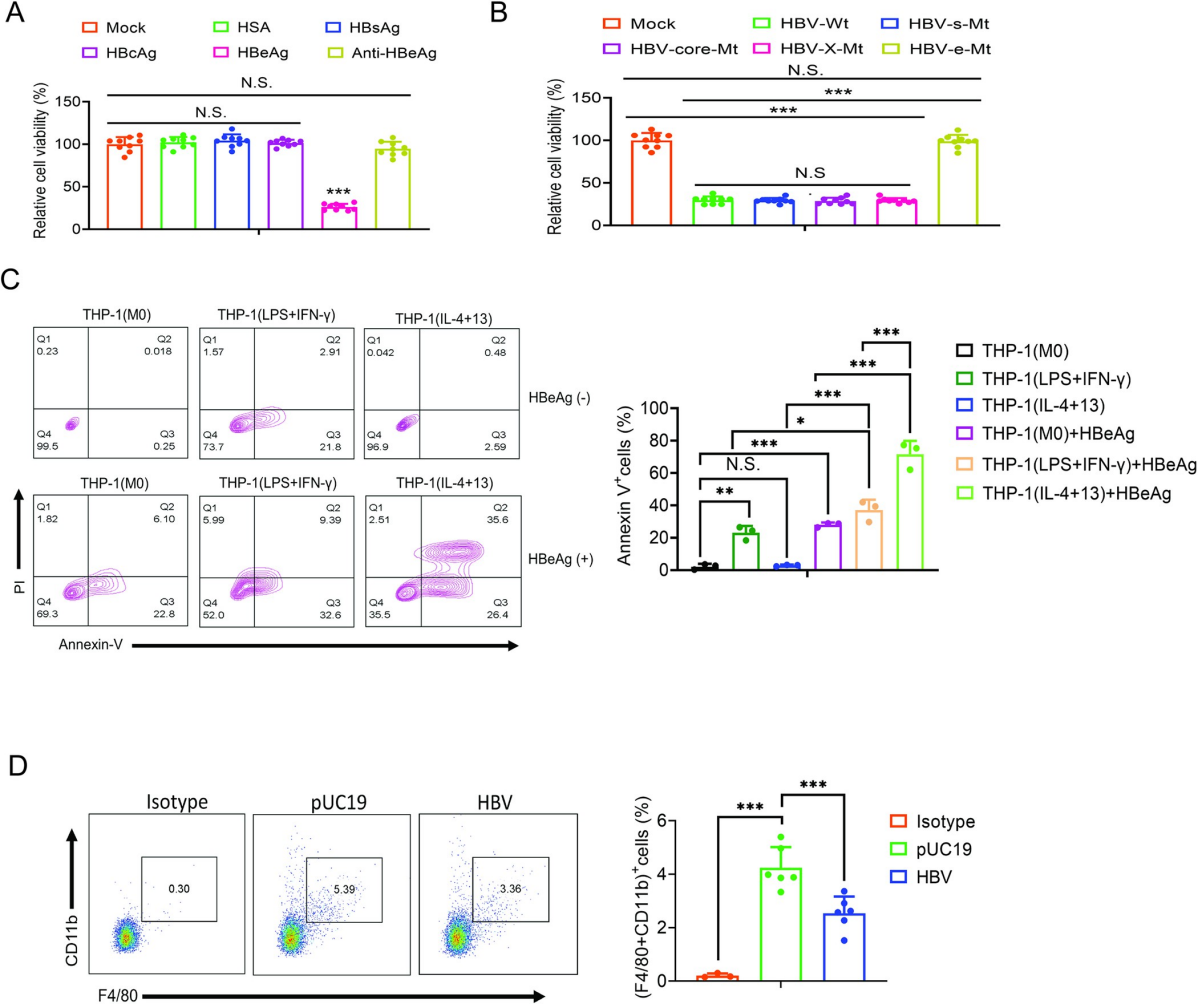
HBeAg differentially induces apoptosis of macrophages. (A) THP-1 macrophages stimulated with HSA, HBsAg, HBcAg, HBeAg together with control IgG or HBeAg in the presence of anti-HBeAg for three days were analyzed for their viability using the CCK8 assay. (B) THP-1 macrophages were stimulated with the incubation medium of Huh7 cells that had been transfected with pUC19 (mock), the 1.3mer wild-type HBV genomic DNA, or the 1.3mer HBV genomic DNA with mutations that abolished the expression of HBsAg, HBcAg, the X protein or HBeAg and then analyzed for their viability using the CCK8 assay. (C) THP-1 M0, M1-like and M2-like macrophages without or with HBeAg treatment were stained for annexin V and with propidium iodide and analyzed by flow cytometry. The statistical analysis of annexin V-positive cells is shown in the bar chart. (D) Kupffer cells were isolated from mice four days after the injection of pUC19 or the HBV genomic DNA and stained for CD11b and F4/80, two markers of Kupffer cells. The staining using the isotype antibody control was also shown. The statistical analysis is shown in the bar chart. In (A-D), N.S., not significant; *, *p*<0.05; **, *p*<0.01; ***, *p*<0.001.

In addition to its ability to regulate OXPHOS, DAP3 can also promote extrinsic apoptosis of cells [12]. To determine whether its induction by HBeAg is associated with the cell death of macrophages, we measured the cell viability of THP-1 macrophages. The treatment of THP-1 M0 macrophages with the control HSA protein, HBcAg or HBsAg had no effect on the cell viability. However, the treatment of THP-1 macrophages with HBeAg greatly reduced the viability of THP-1 macrophages (Fig 5A). This reduction of cell viability was specific to HBeAg, as it was abolished by the antibody directed against HBeAg. Similarly, the viability of THP-1 macrophages was significantly reduced if they were treated with the incubation medium of Huh7 cells that had been transfected with the wild-type HBV genome or the HBV genome that was incapable of expressing HBsAg, HBcAg or the X protein (HBx). However, the cell viability was not reduced by the treatment with the incubation medium of Huh7 cells that had been transfected with the HBV genome that was incapable of expressing only HBeAg (Fig 5B). These results further indicated the importance of HBeAg in the induction of cell death. To understand how HBeAg reduced cell viability, we conducted flow cytometry by staining cells for annexin V, a marker of apoptotic cell death, and cellular DNA with propidium iodide (PI). As a control, we first analyzed control (i.e., M0), M1-like THP-1 macrophages that were treated with LPS+IFN-γ and M2-like THP-1 macrophages that were treated with IL-4+IL-13. As shown in Fig 5C, no apparent apoptotic cell deaths could be detected for M0 or M2-like THP-1 macrophages. However, approximately 20% of M1-like macrophages underwent early apoptosis (i.e., annexin V-positive and PI-negative). When THP-1 macrophages were treated with HBeAg, approximately 30% of M0 macrophages, 40% of M1-like THP-1 macrophages and, surprisingly, more than 60% of M2-like THP-1 macrophages were apoptotic. Similar results were obtained when the cellular DNA content was separately analyzed by flow cytometry. As shown in S5A Fig, M0 and M2-like THP-1(IL4+13) macrophages had no reduced DNA contents whereas nearly 20% of M1-like THP-1(LPS+IFN-γ) macrophages had reduced DNA contents, indicative of DNA degradations. The treatment of these macrophages with HBeAg increased the percentage of cells with reduced DNA contents to slightly over 30% for M0 and M1-like macrophages and nearly 70% for M2-like macrophages. These results were consistent with the results shown in Fig 5A and 5B and indicated that HBeAg could induce the apoptosis of M0 macrophages, enhance the apoptosis of M1-like macrophages, and trigger the apoptosis of the great majority of M2-like macrophages.

The induction of cell death by HBeAg was not specific to THP-1 macrophages. As HBeAg could also reduce the viability of human MDMs, whether they were induced by GM-CSF to undergo the M1-like polarization or by CSF-1 to undergo the M2-like polarization (S5B Fig). We had previously found that Kupffer cells isolated from HBV-negative mice born to naïve mice and those born to hemizygous HBV-transgenic dams would undergo M1-like and M2-like polarizations, respectively, if they were stimulated with HBeAg [6]. We termed those HBV-negative mice born to HBV-positive dams TGD mice, for transgenic-derived mice in brief. As shown in S5C Fig, HBeAg could also reduce the viability of Kupffer cells isolated from either control mice or TGD mice. We had also injected mice with either the control pUC19 vector or the 1.3mer HBV genomic DNA for the confirmation of these *ex vivo* results. As shown in Fig 5D, the analysis of Kupffer cells using CD11b and F4/80 as the markers similarly revealed a reduction of the Kupffer cell population by HBV. These results were also confirmed by the analysis of the Kupffer cell population in transgenic mice that carried the entire HBV genome (i.e., Tg05 mice) or only the precore protein gene (i.e., TgHBe mice). As shown in S5D Fig, the Kupffer cell population in these two groups of transgenic mice was reduced by ~40% when it was compared against the control mice. Taken together, these results indicated that HBV, and more specifically HBeAg, could reduce the population of Kupffer cells both *ex vivo* and *in vivo*.

### DR5 mediates HBeAg-induced apoptosis but not pyroptosis of macrophages

**Fig 6:**
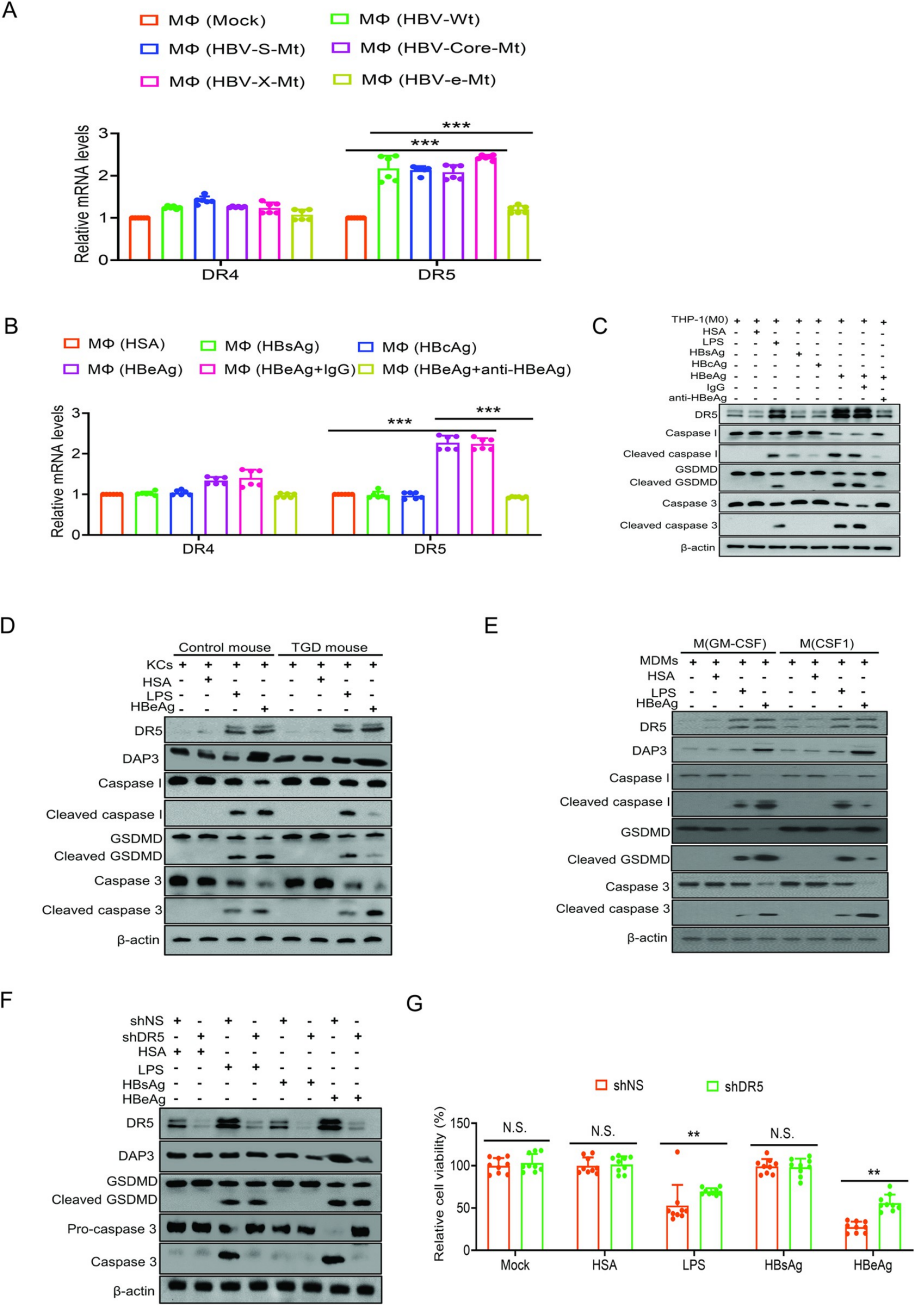
HBeAg induces DR5 to promote apoptosis but not pyroptosis of macrophages. (A) THP-1 macrophages were incubated with the incubation medium of Huh7 cells that had been transfected with pUC19, the 1.3mer HBV genomic DNA or the HBV genomic DNA with mutations that abolished the expression HBsAg, HBcAg or HBeAg followed by RT-qPCR analysis of DR4 and DR5 RNAs. (B) THP-1 macrophages were treated with HSA, HBsAg, HBcAg, HBeAg+control IgG or HBeAg+anti-HBeAg and then lysed for analysis of DR4, DR5 RNAs by RT-qPCR. (C) THP-1 macrophages treated the same way as in (B) were lysed for immunoblot analysis. (D) Kupffer cells isolated from control mice and TGD mice were treated with HSA, LPS or HBeAg and then lysed for immunoblot analysis. (E) The M1-like M(GM-CSF) and the M2-like M(CSF-1) human MDMs were treated with HSA, LPS, or HBeAg and then lysed for immunoblot analysis. (F) THP-1 macrophages transduced with a lentiviral vector that expressed the nonspecific shRNA (shNS) or the DR5 shRNA (shDR5) were stimulated with HSA, LPS, HBsAg or HBeAg and then lysed for immunoblot analysis. (G) THP-1 macrophages without (shNS) or with DR5 silencing (shDR5) were not treated (mock) or treated with HSA, LPS, HBsAg or HBeAg and then analyzed for their viability using the CCK8 assay. In (A), (B) and (G), N.S., not significant; **, *p*<0.01; ***, *p*<0.001.

DAP3 mediates extrinsic apoptosis that is initiated by death receptors (DR) [12]. We had previously conducted an RNA-seq analysis to compare the gene expression profiles of THP-1 macrophages co-cultured with Huh7 cells that were transfected with pUC19 or the 1.3mer HBV genomic DNA [9]. The examination of those data revealed the upregulation of death receptor 5 (DR5) by HBV. As shown in Fig 6A, the treatment of THP-1 macrophages with the incubation medium of Huh7 cells that had been transfected with the wild-type HBV genomic DNA or the HBV DNA mutant that was incapable of expressing HBsAg or HBcAg led to a significant increase of the DR5 expression with only a marginal effect on the DR4 expression. This increase was not observed if THP-1 macrophages were treated with the incubation media of Huh7 cells that had been transfected with the HBV genomic DNA that was incapable of expressing only HBeAg. These results indicated that HBeAg was essential for HBV to induce the DR5 expression in THP-1 macrophages. Indeed, the treatment of THP-1 macrophages with HBeAg, but not other HBV antigens, could also induce the expression of DR5 (Fig 6B). This induction of DR5 expression by HBeAg was specific, as it could be abolished by the antibody directed against HBeAg.

The upregulation of DR5 was also confirmed by immunoblot analysis. As shown in Fig 6C, THP-1 macrophages treated with LPS as well as HBeAg, but not HSA, HBcAg or HBsAg, induced the expression of both long and short isoforms of DR5, which are encoded by two RNA variants [15,16]. The induction of DR5 by HBeAg was specific, as it was abolished by the anti-HBeAg antibody but not by the control antibody. To understand how HBeAg induced the cell death of macrophages, we also analyzed caspase 1, gasdermin D (GSDMD) and caspase 3. Caspase 1 is present in the cell as an inactive zymogen and can undergo autoproteolytic cleavage upon the assembly and the activation of inflammasomes. It can then cleave GSDMD to induce pyroptosis [17]. Caspase 3 is critical for apoptosis and its activation requires the conversion of procaspase 3 to caspase 3. As shown in the same figure, both LPS and HBeAg could induce the cleavage of caspase 1, GSDMD and caspase 3, indicating that both LPS and HBeAg could induce pyroptosis and apoptosis of THP-1 macrophages. In agreement with the RNA results, HBsAg and HBcAg did not increase the protein level of DR5 and induced only a low level of caspase 1 cleavage without a detectable effect on GSDMD and caspase 3. This result was consistent with the result shown in Fig 5A, which revealed little effect of HBsAg and HBcAg on the cell viability. We had also repeated the same experiment using Kupffer cells isolated from control mice and TGD mice, which were HBV-negative mice born to HBV-negative dams and HBV-positive dams, respectively. We had previously shown that Kupffer cells isolated from control mice and TGD mice would undergo M1-like polarization and M2-like polarization, respectively, upon the treatment of HBeAg [6,9]. Indeed, as shown in S6A Fig, the analysis of M1 markers IL-1β, TNF-α and iNOS and M2 markers IL-10, CD163 and Arg-1 confirmed that Kupffer cells isolated from control mice and TGD mice displayed the M1-like and M2-like phenotypes, respectively, after the stimulation with HBV. We then tested the effect of LPS and HBeAg on Kupffer cells isolated from control mice and TGD mice. As shown in Fig 6D, the same as what was observed with THP-1 macrophages, LPS was able to induce the expression of DR5, but not DAP3, and the cleavage of caspase 1, GSDMD and caspase 3 in Kupffer cells isolated from both control and TGD mice. In contrast, although HBeAg was also able to induce the expression of DR5 and DAP3 in control and TGD mice and the cleavage of caspase 1, GSDMD and caspase 3 in Kupffer cells isolated from control mice, its ability to induce the cleavage of caspase 1 and GSDMD was impaired in Kupffer cells isolated from TGD mice with a concomitant enhancement of the cleavage of caspase 3. these results indicated that HBeAg could induce both pyroptosis and apoptosis of M1-like macrophages but primarily only apoptosis of M2-like macrophages. Similar results were obtained with human MDMs. As shown in Fig 6E, LPS induced the expression of DR5 but not DAP3 and the cleavage of caspase 1, GSDMD and caspase 3 in both M(GM-CSF) (i.e., M1-like) macrophages and M(CSF-1) (i.e., M2-like) macrophages. In contrast, although HBeAg could induce DR5 and DAP3 in both M(GM-CSF) and M(CSF-1) macrophages and the cleavage of caspase 1, GSDMD and caspase 3 in M(GM-CSF) macrophages, its ability to cleave caspase 1 and GSDMD in M(CSF-1) macrophages was impaired with the concomitant enhancement of the cleavage of caspase 3. The ability of HBeAg to induce pyroptosis in M1-like macrophages provided an explanation to why the overall viabilities of M1-like and M2-like macrophages were similar after the treatment of HBeAg (S5B, S5C and S6B Figs), in spite of our observation that ~40% of M1-like macrophages and ~60% of M2-like macrophage treated with HBeAg would undergo apoptosis (see Fig 5C and also below).

As DR5 was induced by HBeAg and is known to activate extrinsic apoptosis, we investigated its role in HBeAg-induced apoptosis by silencing its expression in THP-1 macrophages (S6C and S6D Fig). As shown in Fig 6F, the silencing of DR5 had no effect on DAP3, GSDMD and caspase 3 in cells treated with HSA or HBsAg. However, it suppressed the cleavage of caspase 3 without affecting the cleavage of GSDMD in THP-1 macrophages treated with LPS or HBeAg. This DR5 silencing did not affect the DAP3 level of LPS-treated cells, but it abolished the induction of DAP3 induced by HBeAg, indicating that the induction of DAP3 by HBeAg was dependent on DR5. We also measured the viability of THP-1 macrophages. As shown in Fig 6G, the silencing of DR5 partially restored the viability of cells treated with LPS and HBeAg. This result was consistent with the findings mentioned above that LPS and HBeAg induced both apoptosis and pyroptosis and that DR5 silencing suppressed the former without affecting the latter. The silencing of DR5 did not decrease the protein level of IL-1β released from cells induced either by LPS, HBsAg or HBeAg (S6E Fig), confirming its lack of effect on caspase 1 and GSDMD, which mediate the cleavage and the release, respectively, of IL-1β from cells [17].

### HBeAg induces DAP3 via toll-like receptor 4 to metabolically reprogram macrophages

**Fig 7:**
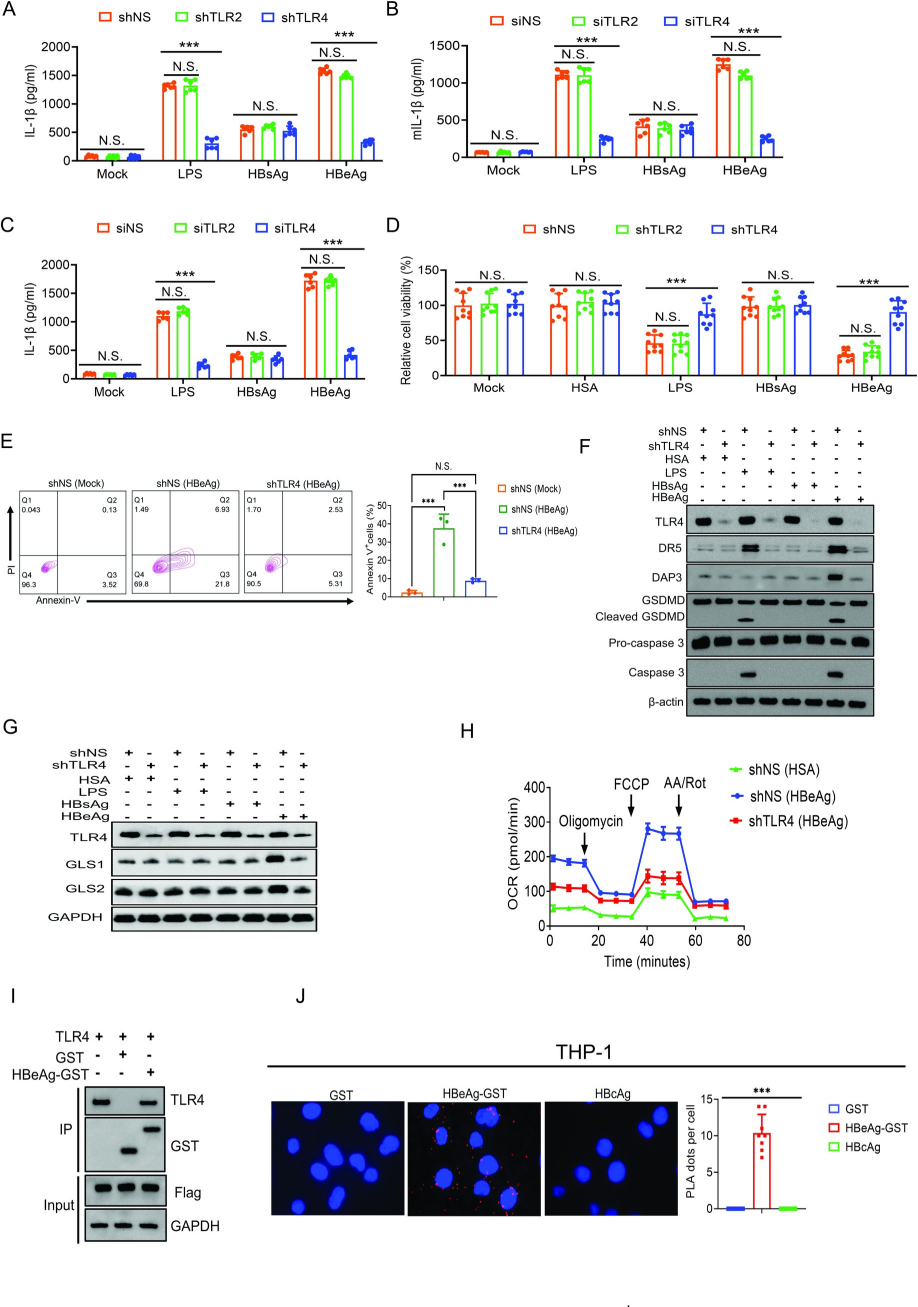
TLR4 mediates the effects of HBeAg on macrophages. (A) THP-1 macrophages transduced by a lentiviral vector that expressed nonspecific shRNA (shNS), TLR2 shRNA (shTLR2) or TLR4 shRNA (shTLR4) were not treated (mock) or treated with LPS, HBsAg or HBeAg. The incubation media were then harvested for the measurement of IL-1β levels by ELISA. (B) Kupffer cells isolated from control mice were treated with nonspecific siRNA (siNS), TLR2 siRNA (siTLR2) or TLR4 siRNA (siTLR4) followed by the treatment with LPS, HBsAg or HBeAg. The incubation media were then harvested for the quantification of mouse IL-1β (mIL-1β) by ELISA. (C) Human MDMs treated with siNS, siTLR2 or siTLR4 were treated with LPS, HBsAg or HBeAg. The incubation media were then harvested for quantification of IL-1β by ELISA. (D) THP-1 macrophages without or with TLR2 or TLR4 silencing were not treated (mock) or treated with HSA, LPS, HBsAg or HBeAg for three days. The viability of cells was then analyzed by the CCK8 assay. (E) THP-1 macrophages without (shNS) or with TLR4 silencing (shTLR4) were not treated (mock) or treated with HBeAg. Cells were then stained with propidium iodide and with the anti-annexin V antibody for the analysis of apoptosis by flow cytometry. (F) THP-1 macrophages without (shNS) or with TLR4 silencing (shTLR4) were treated with HSA, LPS, HBsAg or HBeAg and then lysed for immunoblot analysis. (G) THP-1 macrophages without (shNS) or with TLR4 silencing (shTLR4) were treated with HSA, LPS, HBsAg or HBeAg and then lysed for immunoblot analysis. (H) THP-1 macrophages without (shNS) or with TLR4 silencing (shTLR4) were treated with HSA or HBeAg and then subjected to Seahorse assay for the OCR. (I) HEK293 cells were transfected with the expression plasmid of Flag-tagged TLR4, lysed and treated with GST-HBeAg, and then incubated with glutathione beads for the pull-down experiment. The protein sample was then analyzed by immunoblot using the anti-Flag, anti-GST and anti-TLR4 antibodies. Input represents the total cell lysates prior to the GST-pulldown experiment. GAPDH was used as the loading control. (J) THP-1 macrophages with the treatment of either GST, HBeAg-GST or HBcAg were subjected to the proximity ligation assay (PLA) for the co-localization analysis. Red dots represent positive colocalization signals. DAPI was used to stain the nuclei (blue color). The chart to the right is the average number of PLA signals per cell after a total of 50 cells were analyzed. In (A-E), N.S., not significant; ***, *p*<0.001.

Toll-like receptors (TLRs) play pivotal roles in innate immunity and are expressed in sentinel immune cells such as macrophages and dendritic cells [18]. TLR4 is the receptor for the bacterial endotoxin LPS [19]. As LPS and HBeAg could both induce pyroptosis and apoptosis of M1-like macrophages, we examined whether TLR4 also mediated the effects of HBeAg on macrophages by silencing its expression in THP-1 macrophages (S7A and S7B Fig). We also included TLR2, which could form heterodimers with TLR1 or TLR6 [20], in the analysis. As expected, the silencing of TLR4, but not TLR2, suppressed the release of IL-1β from THP-1 macrophages that were treated with LPS (Fig 7A). Interestingly, the silencing of TLR4, but not TLR2, also greatly suppressed the release of IL-1β from THP-1 macrophages that were treated with HBeAg. Neither TLR2 nor TLR4 silencing had any effect on the release of IL-1β from THP-1 macrophages that were treated with HBsAg. When TLR2 and TLR4 silencing experiments were repeated using mouse Kupffer cells and human MDMs (S7C and S7D Fig), TLR4 was again found to be required for the release of IL-1β from these cell types that were stimulated by LPS and HBeAg but not by HBsAg (Fig 7B and 7C). We also analyzed the effect of TLR4 on HBeAg-induced cell death of THP-1 macrophages. The silencing of TLR4 but not TLR2 largely restored the viability of cells treated with LPS or HBeAg (Fig 7D). It also suppressed apoptosis induced by HBeAg (Fig 7E). These results were confirmed by immunoblot analysis. As shown in Fig 7F, both LPS and HBeAg induced the expression of DR5 and the cleavage of GSDMD and caspase 3 in THP-1 macrophages, and these effects of LPS and HBeAg were abolished by the silencing of TLR4. In agreement with the results shown above, HBeAg also induced the expression of DAP3, which was also abolished by TLR4 silencing. The silencing of TLR4 also reduced the expression of GLS1 and GLS2 induced by HBeAg. However, it had no effect on GLS1/2 in THP-1 macrophages treated with LPS, HSA, HBsAg or HBcAg (Fig 7G). The TLR4 silencing also reduced the OCR of THP-1 macrophages (Figs 7H and S7E). The above results together provided strong evidence that TLR4 mediated the effects of HBeAg, including the induction of programmed cell deaths and OXPHOS, on macrophages.

The critical role of TLR4 in mediating the effects of HBeAg on macrophages raised the possibility that TLR4 might be the elusive receptor for HBeAg. To test this possibility, we expressed TLR4 in HEK293 cells, which allowed efficient expression of recombinant proteins, and conducted a glutathione-*s*-transferase (GST)-pulldown experiment to determine whether HBeAg could bind to TLR4. As shown in Fig 7I, TLR4 could be pulled down by the HBeAg-GST fusion protein but not by the GST control. Due to the difficulty to detect the interaction between TLR4 and HBeAg in THP-1 macrophages, we conducted the highly sensitive proximity ligation assay. As shown in Fig 7J, the colocalization of TLR4 with the HBeAg-GST fusion protein could be detected in THP-1 macrophages. However, such colocalization was not detected when TLR4 was analyzed for its interaction with the GST control protein or HBcAg. The ability of TLR4 to bind to and colocalize with HBeAg and mediate the effects of HBeAg on macrophages supports the argument that TLR4 is the receptor for HBeAg.

## Discussion

M1 and M2 macrophages are known to display distinct metabolic profiles with the former exhibiting low OXPHOS and high glycolytic activities and the latter exhibiting the opposite metabolic activities. In this report, we demonstrated that, although HCV could induce M1-like polarization of macrophages with the typical metabolic profile of low OXPHOS and high glycolysis, HBV induced the M1-like polarization of macrophages with an atypical metabolic profile of high OXPHOS and low glycolysis (Fig 1). We also found that the effect of HBV on macrophages was mediated by HBeAg, which was essential and sufficient to promote OXPHOS and suppress glycolysis of macrophages (Fig 2). In contrast to HBeAg, HBsAg and HBcAg induced low OXPHOS and high glycolytic activities (Fig 2A). This unique ability of HBeAg to induce the atypical metabolism was detected with all of the macrophages analyzed, including THP-1 macrophages, human MDMs and mouse Kupffer cells (Figs 1 and 2).

HBeAg could induce the expression of DAP3 and mitochondrial genes that encode enzymes required for the OXPHOS (Fig 3A and 3B). The induction of mitochondrial genes by HBeAg was likely the outcome of the induction of DAP3 expression, as the silencing of DAP3 reduced the expression of these genes (S3C Fig), leading to the reduction of OXPHOS (Fig 3F and 3G). These results indicated that HBeAg likely induced the expression of DAP3 to promote the expression of mitochondrial enzymes and OXPHOS. The silencing of DAP3 also increased the IL-1β expression (S3C Fig). As IL-1β can directly suppress HBV replication [9], the induction of DAP3 and OXPHOS by HBeAg is thus important for HBV to attenuate this antiviral response of macrophages to promote HBV replication in patients [7].

We had further investigated the effect of HBV on the metabolism of macrophages by conducting the metabolomic analysis. Our results indicated that HBV and HBeAg could significantly increase the glutamate level in macrophages (Fig 4A and 4B). This increase was associated with the increase of GLS1/2 expression (Fig 4E). As the addition of glutamate to the incubation media of HBeAg-treated macrophages enhanced OXPHOS (Fig 4G) and the treatment of macrophages with GLS inhibitors suppressed the OXPHOS induced by HBeAg (Fig 4H), HBeAg apparently induced GLS1/2 to facilitate the conversion of glutamine to glutamate to promote the OXPHOS. Glutamate can be converted to αKG, an important intermediate of the TCA cycle [21]. Our ^13^C-tracing experiment indeed confirmed that glutamine in HBeAg-treated macrophages was converted to glutamate, αKG and other metabolic intermediates of the TCA cycle (S4C Fig). In contrast, little conversion of glutamine to αKG was observed in mock-treated macrophages. Thus, in addition to inducing the expression of DAP3 and mitochondrial genes to promote OXPHOS, HBeAg also induced GLS to increase the pool of glutamate to promote OXPHOS. These results also provided an explanation to why glutamate could suppress IL-1β expression only in the presence of HBeAg (Fig 4I), as glutamate could only be efficiently converted to αKG in the presence of HBeAg to promote OXPHOS, which was associated with the reduction of IL-1β production (Fig 3A and 3B).

An interesting discovery of our work was the ability of HBeAg to induce both pyroptosis and apoptosis in M1-like macrophages and primarily apoptosis in M2-like macrophages both *in vitro* and *in vivo* (Figs 5 and S5). The induction of pyroptosis of M1-like macrophages by HBeAg was apparently due to the activation of inflammasomes, which led to the self-cleavage of caspase 1 and its subsequent cleavage of GSDMD and IL-1β to trigger pyroptosis and the release of IL-1β (Fig 6C and 6D). In contrast, the induction of apoptosis by HBeAg was due to the induction of DR5, which led to the activation of caspase 3 and cell death (Fig 6E). DR5 can be activated by the binding of its ligand tumor necrosis factor-related apoptosis-inducing ligand (TRAIL) or by its over-expression. This will lead to the formation of the death-inducing signaling complex (DISC) and the activation of caspases [22]. It is conceivable that the increased expression of DR5 induced by HBeAg activates this extrinsic apoptotic pathway. Note that HBeAg induced cell deaths in a fraction but not all of macrophages including Kupffer cells. This is perhaps the reason why a previous study found the accumulation of M2-like macrophages in the liver of chronic HBV patients [23]. M2 macrophages secrete TGF-β and the anti-inflammatory cytokine IL-10, which are important for the initiation of tissue repair and the resolution of the inflammatory response [24]. Why HBeAg induced the death of only a fraction of macrophages including Kupffer cells is an interesting question. This may be due to the plasticity and the heterogeneity of these cells. The observation that Kupffer cell populations were reduced in transgenic mice that carried either the entire HBV genome or only the precore protein gene also supported the notion that HBeAg could induce Kupffer cell deaths *in vivo* (S5D Fig). It will be interesting to determine how this reduction of Kupffer cells by HBeAg affects HBV pathogenesis during chronic infection.

The receptor for LPS is TLR4. Our results indicated that TLR4 was also essential for HBeAg to induce IL-1β (Fig 7A–7C), pyroptotic and apoptotic cell deaths (Fig 7D–7F), GLS1/2 (Fig 7G) and the OXPHOS (Fig 7H). TLR4 could also bind to and colocalize with HBeAg (Fig 7I and 7J). As TLR4 can physically bind to HBeAg and is essential for HBeAg to regulate the metabolism and the programmed cell deaths of macrophages, TLR4 is likely also the receptor for HBeAg. It should be noted that, although HBeAg induced DAP3 via DR5, LPS induced DR5 without the induction of DAP3 (Fig 6D–6F). Thus, if TLR4 is also the receptor of HBeAg, as our results suggest, there are significant differences between the signaling pathways activated by LPS and HBeAg. The binding of LPS to TLR4 requires myeloid differentiation 2 (MD2) and can be enhanced by LPS-binding protein (LBP), which then binds to CD14 to interact with TLR4 [25]. It is possible that HBeAg does not interact with LBP and activates a different signaling pathway that leads to the induction of DR5, DAP3 and OXPHOS. In this regard, it is likely that the signaling pathways activated by HBeAg in M1-like and M2-like macrophages are also different, as HBeAg induced both pyroptosis and apoptosis of the former but primarily apoptosis of the latter. Further studies on HBeAg-activated signaling pathways will likely generate many more interesting findings. Our results also indicated that HBsAg and HBcAg, two different viral antigens of HBV, could activate macrophages with a metabolic profile similar to that of M1 macrophages (Fig 2A) without inducing notable cell death (Fig 5A). The receptors for HBsAg and HBcAg were not explored in our study, although it had been suggested that HBsAg could bind to CD14 [26] and HBcAg could interact with TLR2 [27].

Our studies described in this report revealed an unusual activity of HBeAg. This HBV antigen could rewire the metabolism of M1-like macrophages to promote OXPHOS via TLR4, which increased the expression of DAP3 and GLS1/2. DAP3 enhanced the expression of mitochondrial enzymes, and GLS1/2 mediated glutaminolysis to increase the level of glutamate, which was further converted to αKG (S4C Fig). Their combined activities promoted the OXPHOS. This induction of OXPHOS by HBeAg suppressed the induction of IL-1β and was important for HBV to attenuate this antiviral response. In addition, HBeAg could also induce programmed cell deaths of both M1-like and M2-like macrophages. The induction of pyroptotic cell death of M1-like macrophages is likely the outcome of the activation of inflammasomes. Although the clinical consequence for the induction of apoptosis of a high percentage of M2-like macrophage is unclear, it likely plays an important role in HBV pathogenesis during chronic HBV infection.

## Materials and methods

### Ethics statement

The animal work described in this manuscript was approved by the Institutional Animal Care and Use Committee (IACUC) at the University of Southern California (protocol number 20627). All experiments were carried out in compliance with the IACUC regulations for the humane care and use of animals. The human peripheral blood mononuclear cells were obtained from anonymous healthy blood donors with the approval of the Institutional Review Board of the City of Hope.

### Cell lines

The human hepatoma cell line Huh7 was maintained in Dulbecco’s modified essential medium (DMEM) supplemented with 10% fetal bovine serum (FBS). The human embryonic kidney cell line HEK293T was also maintained in DMEM containing 10% FBS. THP-1 cells were human monocytic cells. They were maintained in the RPMI medium containing 10% FBS. Huh7 cells might be transfected with the 1.3mer HBV genomic DNA, its mutants or the control vector pUC19 for two days. Alternatively, they might also be infected by a cell culture-adapted variant of the HCV JFH1 strain (M.O.I. = 0.5–1) for two days [28]. The cell culture media, which contained HBV or HCV, would then be used to treat macrophages.

### Recombinant proteins

The recombinant protein human serum albumin (HSA) (Cat# 10968-HNAY) and GST (Cat#11213-HNAE) were purchased from Sino Biological. HBsAg (Cat# ab91276), HBcAg (Cat# ab49013), and GST-HBeAg (Cat# ab91273) were purchased from Abcam. HBeAg was prepared in our laboratory as previously described [9].

### Differentiation and activation of THP-1 monocytes

THP-1 monocytes maintained in the RPMI medium containing 10% FBS were treated with 100 nM phorbol 12-myristate 13-acetate (PMA) for 48 hours to induce their differentiation into macrophages. The differentiated THP-1 macrophages were then incubated in PMA-free RPMI medium containing 10% FBS for another 24 hours. For the induction of M1-like polarization, THP-1 macrophages were further treated with 20 ng/ml of recombinant human interferon-γ (IFN-γ) and 10 ng/ml of lipopolysaccharides (LPS) for another 48 hours. For the induction of M2-like polarization, THP-1 macrophages were treated with 20 ng/ml of recombinant human interleukin 13 (IL-13) and 20 ng/ml of recombinant human IL-4 for 48 hours.

### Isolation of human monocytes

Human peripheral blood mononuclear cells (PBMCs) were isolated from healthy donors using the Ficoll gradient (Histopaque-1077, Sigma) as previously described [29]. Briefly, an equal volume of phosphate-buffered saline (PBS) was mixed with the heparin-treated blood, which was then overlaid very slowly on 15 mL Ficoll solution followed by centrifugation at 400x*g* at 22°C with the brake off for 45 minutes. After the removal of the plasma on the top of the gradient, the buffy coat, which contained mononuclear cells and was located at the interface between the plasma on the top and Ficoll at the bottom, was transferred to a 50 mL conical tube, mixed with 30 mL PBS, and then centrifuged at 400x*g* at 22°C for 10 minutes. PBMCs in the pellet were resuspended in 10 mL PBS. Classical monocytes were then purified from freshly isolated PBMCs using the Monocyte Isolation Kit II (Miltenyi Biotec) following the manufacturer’s instructions.

### Human monocytes differentiation and activation

Human monocytes were induced to undergo M1-like or M2-like polarization as previously described [30,31]. Briefly, human monocytes were treated with 50 ng/ml of GM-CSF (R&D) or 50 ng/ml of CSF-1 (Peprotech) for their M1-like or M2-like differentiation, respectively, at 37°C for seven days in a 5% CO_2_ incubator. These monocyte-derived macrophages (MDMs) were then activated with 10 ng/ml of lipopolysaccharide (LPS) for 3 hours at 37°C, rinsed with PBS and incubated in the fresh medium for another 24 hours before the experiments.

### Mice

The Tg05 HBV transgenic mouse line carried the 1.3mer overlength HBV genomic DNA and had a serum HBV titer of nearly 10^9^ genome copies per ml. It was used to generate TGD mice as previously described [6]. Briefly, female hemizygous Tg05 mice were crossed to naïve male mice to generate the HBV-negative mouse pups. These HBV-negative mouse progenies were termed TGD mice. Control mice used in our studies were born to HBV-negative dams. All of the mice used in our studies had the C57BL/6 genetic background.

### Hydrodynamic injection and the isolation of Kupffer cells from mice

Hydrodynamic injection of pUC19 or the 1.3mer HBV genomic DNA that was cloned in the pUC19 vector was performed on 8-10-week-old mice via the tail vein. The DNA was mixed with PBS in a volume that was approximately 8% of the body weight and the injection was completed in 5–8 seconds. Due to the sudden influx of a large volume of saline, the liver would expand to result in the entry of DNA into mouse hepatocytes. The isolation of mouse Kupffer cells was conducted as previously described at 4 days after DNA injection [32]. Briefly, the mouse liver was perfused via the hepatic portal vein first with 50 mL of the albumin solution and next with 30 mL of the solution containing collagenase IV at a flow rate of 7 mL/min. The solutions were maintained at 37°C in a water bath. The perfused liver was then removed from mice and placed in a petri dish containing 35 mL PBS. Liver cells were dispersed with the help of a tweezer, filtered with a 70 μm nylon cell strainer and centrifuged in a 50 mL tube at 60x*g* for 2 minutes. The supernatant, which contained nonparenchymal cells (LNPCs), was transferred to a 50 ml tube and then centrifuged at 583x*g* for 8 minutes at 18°C–25°C. The pellet was resuspended in 5.5 mL DMEM, overlaid on a step gradient consisting of OptiPrep Density Gradient Media with densities of 1.035 (1.5 mL), 1.042 (1.5 mL), 1.056 (1.5 mL) and 1.081 (2 mL) and then centrifuge at 18°C–25°C at 21,400x*g* for another 20 min using a Beckman SW40Ti rotor. Kupffer cells located at the interface of densities 1.042 and 1.056 were isolated and pelleted at 583x*g* for 8 minutes.

### Analysis of cell viability and cell cycle

For the measurement of the cell viability, 50,000–100,000 cells were seeded in each well of the 96-well plate. The cell viability was then assessed using Cell Counting Kit-8 (Dojindo, # CK04-11) following the manufacturer’s instructions. The experiments were conducted in triplicate. For the cell cycle (i.e., DNA content) analysis, cells were resuspended in Krishan’s reagent (0.05 mg/ml propidium iodide, 0.1% trisodium citrate, 0.02 mg/ml ribonuclease A and 0.3% NP-40) and incubated at 37°C for 30 minutes. The cell cycle (i.e., the cellular DNA content) was analyzed by flow cytometry.

### Apoptosis analysis

The apoptosis of cells was analyzed using Annexin V-FITC Apoptosis Staining/Detection Kit (Abcam, cat# ab14085) following the manufacturer’s protocol with some modifications. Briefly, 1x10^5^ THP-1 macrophages were pelleted by centrifugation and then resuspended in 500 μl 1X Annexin V Binding Buffer. After the addition of 5 μl Annexin V-FITC and 5 μl propidium iodide, cells were incubated at room temperature for 5 minutes in the dark followed by flow cytometry analysis.

### Flow cytometry

The flow cytometry was carried out using the BD Cell Analyzer (BD Bioscience), and the data were analyzed using FlowJo 10 software (BD Bioscience). The analysis of Kupffer cells and LNPCs was performed as described previously with some modifications [6]. Briefly, cells were collected and incubated with primary antibodies that might include IgG2a-PE, IgG1-APC, APC anti-human CD11b antibody (Biolegend, #301310), and/or F4/80-PE (eBioscience, #12-4801-82) at 4°C for 30 minutes. Cells were then rinsed with PBS three times followed by flow cytometry analysis.

### Immunoblot analysis

For immunoblotting, cells were chilled on ice, rinsed with ice-cold PBS twice and lysed with RIPA buffer (Sigma-Aldrich, R0278) plus the protease inhibitor cocktail (Thermo Scientific, A32963). Protein concentrations were determined using the BCA Protein Assay Kit (BioRad). About 25 μg protein per sample was loaded on a 10% SDS-PAGE gel and transferred to the PVDF membrane, which was washed with PBS Tween 20 buffer (PBST) (ThermoFisher Scientific, #28360) for 5 minutes three times, blocked with 5% milk in PBST, and incubated with the anti-DAP3 (Abcam, #ab11928), anti-MT-CO1(Abcam,# ab14705), anti-MT-ND1(Abcam,# ab181848), anti-β-actin (Cell signaling technology,# 2146S), anti-DR5 (Cell signaling technology,#8074T), anti-cleaved caspase I (Cell signaling technology,# 2225T), anti-GSDMD (Abcam,# ab219800), anti-cleaved caspase 3 (Cell signaling technology, # 9661S), or the antibody directed against denatured core protein in the cold room overnight. The anti-core antibody was prepared in our lab using the recombinant core protein [33]. After washing with PBST for 10 minutes three times, the membrane was incubated with the horseradish peroxidase-conjugated secondary antibody (Abcam) at room temperature for one hour. The membrane was washed with PBST for 10 minutes three more times and subjected to chemiluminescent analysis. All experiments were repeated at least three times.

### Enzyme-linked immunosorbent assay (ELISA)

For the analysis of IL-1β, the culture medium was diluted 4-fold, and 100 μL of diluted samples were used for ELISA. The ELISA kits for both human IL-1β (#DLB50) and mouse IL-1β (#AF-401-SP) were purchased from R&D Systems. All experiments were repeated at least three times.

### RNA extraction, cDNA synthesis and qPCR

Total RNA from macrophages were isolated using TRIzol reagent (Thermo Fisher Scientific, #15596026) following the manufacturer’s instructions. 200 ng total RNA was used for cDNA synthesis in a 10 μL reaction volume using QuantiTect Reverse Transcription Kit (QIAGEN, #205314). The quantitative PCR (qPCR) was performed using PCR Master Mix (ThermoFisher, #4367659) and StepOne Plus Real-Time PCR system. The housekeeping genes glyceraldehyde 3-phosphate dehydrogenase (GAPDH) or β-actin was used as internal controls. Each qPCR reaction was conducted in triplicate. All of the experiments were repeated at least three times.

### Seahorse assay

THP-1 macrophages, human MDMs, and Kupffer cells with different treatments for 48 hours were subjected to Seahorse assay using the XFe96 Extracellular Flux Analyzer (Agilent) and our previous procedures [9]. Briefly, approximately 0.5x10^5^ cells were seeded in each well of the XF96 plate. After the attachment of macrophages, cells were rinsed twice with fresh media to remove dead (i.e., nonadherent) cells. The oxygen consumption rate (OCR) was measured using the mitochondrial stress test kit. The final results were then normalized against the protein concentrations of the adherent cells. The OCR was then measured in Seahorse XF Base DMEM containing 10 mM glucose, 2 mM L-glutamine, and 1 mM sodium pyruvate under basal conditions and with the addition of 2 μM oligomycin, 0.75 μM fluoro-carbonyl cyanide phenylhydrazone (FCCP) and 0.5 μM rotenone/antimycin A (Rot/AA). The extracellular acidification rate (ECAR) was measured using the glycolytic rate assay kit. The ECAR was measured in Seahorse XF Base Medium with the addition of 5 μM Rot/AA and 500 mM 2-deoxyglucose (2-DG). Oligomycin inhibits the electron flow through the electron transport chain (ETC), resulting in the reduction of mitochondrial respiration (i.e., OCR). FCCP disrupts mitochondrial membrane potential to allow electron flow through the ETC, optimizing the oxygen consumption. Rot and AA are ETC complex I and complex III inhibitors, respectively. Their combined use shut down mitochondrial respiration and enabled the calculation of non-mitochondrial respiration. 2-DG is a glucose analog that inhibits glucose hexokinase and the glycolytic pathway. In Mito Stress Test, time zero indicates the start of the measurement of the basal respiratory rate of the cells. In Glycolytic Rate Assay, time zero indicates the start of the measurement of the basal glycolytic rate of the cells. Each of the basal levels was measured for 15–20 minutes before the drug was injected for the next measurement.

### Gene silencing and protein expression

The procedures for the preparation of lentiviral vectors that expressed different shRNAs or proteins were performed as described previously with some modifications [9,34]. Lentivirus particles were prepared using the plasmid pLKO.1-shRNA scrambled, pLKO.1-shDAP3 (Sigma-Aldrich, #TRCN0000229949), pLKO.1-shDR5 (Sigma-Aldrich, # TRCN0000005930), pLKO.1-TLR2 (Sigma-Aldrich, # TRCN0000057018), pLKO.1-TLR4 (Sigma-Aldrich, # TRCN0000056894) or pCDH-CMV-MCS-EF1α-GreenPuro (SBI, #CD513-B1). pCDH-DAP3 were generated by inserting the DAP3 cDNA fragment, which was isolated from the DAP3 cDNA plasmid (Sino biological, # HG15768-NH), into the XbaI and BamHI restriction enzyme sites of the pCDH-CMV-MCS-EF1α-GreenPurovector. Lentiviral particles were produced by co-transfecting the plasmids mentioned above with the packaging vectors pMD2.G (0.5 μg), pMDLg/pRRE (0.3 μg), and pRSV-Rev (0.7 μg) into HEK293T cells in a 60-mm petri dish using X-tremeGENE HP DNA Transfection Reagent (Sigma-Aldrich, #6366236001). Lentiviral particles were harvested and concentrated with PEG-it virus precipitation solution (System Biosciences, # LV810A-1) for 48 or 72 hours and used to infect target cells in the presence of 4 μg/ml polybrene (Sigma-Aldrich, #H9268). Cells were transduced with lentiviral particles twice and then selected with1 μg/mL puromycin (Sigma-Aldrich, #P8833) for at least two passages. For cells that were transduced by lentiviral particles, they were further selected by flow cytometry based on the expression levels of green fluorescence protein (GFP). Only the top 10% of cells that expressed high levels of GFP were selected. siRNAs targeting human TLR2 (Sigma, SASI_Hs01_00074934), human TLR4 (Sigma, # SASI_Hs01_00122250), mouse TLR2 (Sigma, # SASI_Mm01_00135214), mouse TLR4 (Sigma, # SASI_Mm01_00139037), and siRNA Universal Negative Control (Sigma, #SIC001-10NMOL) were purchased from Sigma-Aldrich. The transfection of siRNA into human MDMs or Kupffer cells was performed using Lipofectamine RNAiMax (Invitrogen) or Lipofectamine 2000 (Invitrogen) following the manufacturers’ protocols. Briefly, human MDMs or Kupffer cells seeded in 6-well plates were transfected with 100 pmole siRNA for 6 hours. The transfected cells were further incubated in fresh media for 18 hours prior to the treatment with HBV antigens.

### Metabolomic and metabolic flux analysis

THP-1 macrophages were seeded in a 6-well dish (~2x10^6^ cells per well) and incubated in RPMI medium for different treatments. For metabolomic analysis, cells were treated with LPS+IFN-γ, IL-4+IL-13, HBV or HCV for 48 hours. HBV and HCV were harvested from the incubation media of Huh7 cells that had been infected by HCV or transfected with HBV genomic DNA or the control vector pUC19 for 48 hours. For metabolic flux analysis, THP-1 macrophages were cultured in RPMI medium that lacked glucose, serine, and glycine but was supplemented with 5% dialyzed FBS (Gibco, A3382001). After a 24-hour pre-treatment with or without 1 μg/mL HBeAg, the medium was replaced with the fresh medium that contained 25 mM [U-^13^C]glucose (Cambridge isotope laboratories, CLM-1396) or 2 mM [U-^13^C]glutamine (Cambridge isotope laboratories, CLM-1822) with or without 1 μg/mL HBeAg for 24 hours. For both metabolomic analysis and metabolic flux analysis, cells were rinsed once with ice-cold PBS after the treatments and then treated with 300 μL 80% methanol that had been stored at -80°C for two hours. Cells were scraped off the plate using the 1 mL micropipette tip, transferred to a 2 mL screw-cap tube (Sarstedt Inc, #72.693.005) and incubated at -80°C overnight. Zirconia/Silica beads 0.1 mm in size were added into the sample in the 2 mL screw-cap tube. Cells were broken thoroughly using the bead-based homogenizer (Bertin Technologies) at 6,800 rpm for 4x30 seconds, and the cell lysates were centrifuged at 15,000 x g for 10 minutes at 4°C. The supernatant was transferred to a new 1.5 mL microcentrifuge tube and dried completely using nitrogen blower or vacuum freeze-dryer. The sample was stored at -80°C prior to analysis by liquid chromatography-mass spectrometry (LC-MS). For the LC-MS analysis, metabolites were mixed with acetonitrile containing 0.2% folic acid solution and centrifuged at 12,000 rpm for 10 min at 4°C. The supernatant was injected into Agilent Accurate Mass 6230 Time of Flight (TOF) coupled with Agilent 1290 liquid chromatography system. Detected ions were deemed metabolites on the basis of unique accurate mass-retention time identifiers for masses exhibiting the expected distribution of accompanying isotopologues [35]. The abundance of metabolites was extracted using Profinder B.08.00 software (Agilent Technologies) with a mass tolerance of <0.005 Da, and the data were normalized against the protein concentrations.

### GST-pull down experiment

1x10^6^ HEK293T cells were seed in each well of a 6-well plate and transfected with the DNA plasmid (4 μg) that expressed the Flag-tagged human TLR4 (Sino Biological, #HG10146-NF) using lipofectamine following the manufacturer’s instructions. Cells were rinsed with ice-cold RNase-free PBS two days after the DNA transfection and lysed with 200 μL lysis buffer (25 mM HEPES, pH7.9, 150 mM NaCl, 0.5 mM EDTA, 10% glycerol,1% Triton X-100, 1 mM DTT and 1:100 dilution of the protease inhibitor cocktail). Cells were further disrupted by passing through a 21G needle 3–5 times and rocked for 20 minutes at 4°C. Cell lysates were centrifuged at 15,000x*g* for 10 mins at 4°C for the removal of cell debris. The supernatant was collected, and additional lysis buffer was added to increase the volume to 1100 μL. 10 μg GST-HBeAg or the control GST was added to 500 μL of cell lysates and the sample was rocked overnight at 4°C. 100ul of the lysates were used as the input control. 20 μL of 5X lysis buffer and 10 μL of 1M DTT were added to the input control and the sample was heated at 95°C for 5 minutes and then stored at -80°C. 25 μL glutathione beads, which had been washed twice with 1 mL ice-cold lysis buffer, were added to the sample. The sample was incubated at room temperature for 2 hours with rocking. The beads were separated from the supernatant using a magnetic separator and washed twice with the lysis buffer. After the 2nd wash, the content was transferred to a new 1.5ml tube and washed three more times. The beads were then resuspended in 100 μL 1X Laemmli buffer and heated at 95°C for 5 minutes. After the removal of the beads with a magnetic separator, the supernatant was transferred to a new tube. 10 μL of the protein sample and the input control were subjected to electrophoresis in a sodium dodecyl sulfate-polyacrylamide gel (SDS-PAGE) and transferred onto the Immobilon-P PVDF membrane (Merck Millipore, Billerica, MA, USA). After blocking with 5% skim milk in phosphate-buffered saline with 0.1% Tween-20 for 1 hour, the membrane was incubated with the antibodies and analyzed using Enhanced Chemiluminescence (ECL) system (Bio-Rad, Hercules, CA, USA).

### Proximity ligation assay

The proximity ligation assay (PLA) was conducted using the Duolink PLA In Situ Red Starter Kit from Sigma-Aldrich (cat. # DUO92101-1KT). THP1 cells differentiated into macrophages with PMA were incubated with GST (10 μg/mL), GST-HBeAg (10 μg/mL) or HBcAg (10 μg/mL) for 1 hour at 4°C. Cells were then fixed with -20°C acetone for 2 minutes at room temperature. After blocking with PBS containing 1% BSA, cells were incubated with TLR4 and GST primary antibodies overnight at 4°C. The subsequent steps of the proximity ligation assay (PLA) were then conducted following the manufacture’s protocol.

### Quantification and statistical analysis

All experiments including ELISA, qPCR, Seahorse analysis, PLA and mouse studies were conducted in triplicate and repeated at least three times unless otherwise specified. The results shown represented mean±SEM. Statistical analyses were conducted using Prism software (GraphPad 8). To determine statistical significance, we employed two-tailed unpaired Student’s t-tests and Mann-Whitney tests for comparing two groups, and ANOVA tests for comparing more than two groups. We considered *p*< 0.05 statistically significant. Source data for quantitative analyses are shown in S1 Data, and the original uncropped immunoblot results are shown in S2 Data.

## Supporting information

S1 Fig Analysis of M1-like and M2-like markers of THP-1 macrophages and human MDMs and quantitative analysis of the metabolic parameters of OCR and ECAR. (A) THP-1 macrophages (THP-1(M0)) were treated with LPS and IFN-γ (THP-1(LPS+IFN-γ), with IL-4 and IL-13 (THP-1(IL-4+13)), with the incubation medium of Huh7 cells that had been infected with HCV (THP-1(M0)+HCV) for 24 hours, or with the incubation medium of Huh7 cells that had been transfected with the 1.3mer HBV genomic DNA (THP-1(M0)+HBV) for 48 hours. Cells were then lysed for the analysis of IL-1β, TNF-α, iNOS, CD163, IL-10 and MRC-1 RNAs by RT-qPCR. (B) Human MDMs were treated with GM-CSF (M(GM-CSF) or CSF-1 (M(CSF-1) for the induction of M1-like or M2-like polarization, respectively. In addition, M(GM-CSF) were also treated with HCV or HBV. Cells were then lysed for analysis of IL-1β, TNF-α, iNOS, CD163, IL-10 and MRC-1 RNAs by RT-qPCR. (C-D) and (E-F) are the quantitative analyses of the metabolic parameters of OCRs and ECARs of Fig 1A and 1B, respectively. N.S., not significant; *, *p*<0.05; **, *p*<0.01; ***, *p*<0.001.
(TIF)

S2 Fig Metabolic parameters of OCR and ECAR shown in Fig 2 and dose-dependent effect of HBeAg on the OCR of THP-1 macrophages. (A-B), (D-E) and (F-G) are the quantitative analyses of the metabolic parameters of OCRs and ECARs of Fig 2A–2C, respectively. (C) Analysis of the OCR of THP-1 macrophages that were treated with 0.25, 0.5 or 1 μg/mL of HBeAg for 48 hours. THP-1 macrophages not treated (Mock) was used as the control. N.S., not significant; *, *p*<0.05; **, *p*<0.01; ***, *p*<0.001.
(TIF)

S3 Fig Effect of DAP3 silencing on the expression of mitochondrial genes and metabolism in THP-1 macrophages. (A) RT-qPCR analysis of relative DAP3 RNA levels in THP-1 macrophages with (shDAP3) and without (shNS) DAP3 silencing. The level of DAP3 RNA without silencing was arbitrarily defined as 1. (B) Immunoblot analysis of DAP3 in THP-1 macrophages with and without DAP3 silencing. (C) The relative RNA levels of mitochondrial genes, IL-1β and DAP3 in THP-1 macrophages with and without DAP3 silencing and with and without HBV treatment were determined by RT-qPCR. (D), (E) and (G) are the metabolic parameters of the OCR of THP-1 macrophages shown in Fig 3F–3H, respectively. (F) THP-1 macrophages with and without DAP3 silencing were treated with 1 μg/mL HBeAg for 48 hours and then subjected to Seahorse analysis for ECAR. N.S., not significant; *, *p*<0.05; **, *p*<0.01; ***, *p*<0.001.
(TIF)

S4 Fig Analysis of the effect of glutamate on the OCR of THP-1 macrophages and the ^13^C-tracing experiments. (A) and (B) are the quantitative analyses of individual metabolic parameters of the OCR studies shown in Fig 4G and 4H, respectively. N.S., not significant; *, *p*<0.05; **, *p*<0.01; ***, *p*<0.001. (C) The [U-^13^C]glutamine-tracing study on THP-1 macrophages with and without HBeAg treatment.
(TIF)

S5 Fig Effects of HBeAg on apoptosis and viability of macrophages. (A) THP-1 M0, M1-like and M2-like macrophages with and without the treatment of HBeAg were stained with propidium iodide and then analyzed by flow cytometry. (B) Human MDMs treated with GM-CSF for the induction of M1-like polarization or with CSF-1 for M2-like polarization were analyzed for their viability using the CCK8 assay. (C) Kupffer cells isolated from control mice (Cont KC) or HBV-negative mice born to hemizygous HBV transgenic dams (TGD KC) were analyzed for their viability using the CCK8 assay. (D) Kupffer cells were isolated from control mice and HBV transgenic mice carrying either the 1.3mer HBV genome (Tg05) or the precore protein gene (TgHBe) as described in the Methods section for analysis. Both Tg05 and TgHBe mice had reduced numbers of Kupffer cells. In (B), (C) and (D), N.S., not significant; **, *p*<0.01; ***, *p*<0.001.
(TIF)

S6 Fig Effects of HBeAg on mouse Kupffer cells and THP-1 macrophages and DR5 silencing. (A) Kupffer cells isolated from control mice or TGD mice that had been injected with pUC19 (HBV-) or the 1.3mer HBV genomic DNA (HBV+) were analyzed for the RNA levels of M1 markers (i.e., IL-1β and TNF-α) and M2 markers (i.e., IL-10 and CD163) by RT-qPCR. Control mice intraperitoneally injected with LPS (1 mg/kg) for 48 hours were also analyzed to serve as the control. (B) THP-1 macrophages without (M0) or with induction of M1-like polarization (THP-1(LPS+IFN-γ)) or M2-like polarization (THP-1(IL4+13)) and without (mock) or with the treatment of HSA or HBeAg were analyzed for their viability using the CCK8 assay. (C) THP-1 macrophages without (shNS) or with DR5 silencing (shDR5) were lysed for the analysis of DR5 RNA by RT-qPCR. (D) THP-1 macrophages as described in (C) were lysed for immunoblot analysis of DR5. (E) The incubation media of THP-1 macrophages without (shNS) or with DR5 silencing (shDR5) and without (mock) or with the treatment of LPS, HBsAg or HBeAg were collected and analyzed for the level of IL-1β using ELISA. In (A-C) and (E), N.S., not significant; *, *p*<0.05; **, *p*<0.01; ***, *p*<0.001.
(TIF)

S7 Fig Analysis of the effects of TLR2 and TLR4 silencing on macrophages. (A) THP-1 macrophages with shNS, shTLR2 or shTLR4 silencing were analyzed for the RNA level of TLR2 and TLR4 by RT-qPCR. (B) Immunoblot analysis of THP-1 macrophages with shNS, ShTLR2 or shTLR4 silencing. (C) Kupffer cells treated with control siRNA (siNS), TLR2 siRNA (siTLR2) or TLR4 siRNA (siTLR4) were lysed for analysis of mouse TLR2 (mTLR2) and TLR4 (mTLR4) RNAs by RT-qPCR. (D) Human MDMs treated with siNS, siTLR2 or siTLR4 were lysed for RNA analysis by RT-qPCR. (E) Quantitative analysis of the metabolic parameters of the OCR study shown in Fig 7H. In (A) and (C-E), N.S., not significant; *, *p*<0.05; **, *p*<0.01; ***, *p*<0.001.
(TIF)

S1 Data Source data for statistical analyses in this paper. (XLSX)

S2 Data Original immunoblot results shown in this paper. (PDF)
