# Supplementary figures and images for: Hepatitis B virus e antigen induces atypical metabolism and differentially regulates programmed cell deaths of macrophages
Source: PLoS Pathog. 2024 Mar 11;20(3):e1012079. doi: 10.1371/journal.ppat.1012079 (PMC10957081; doi:10.1371/journal.ppat.1012079)

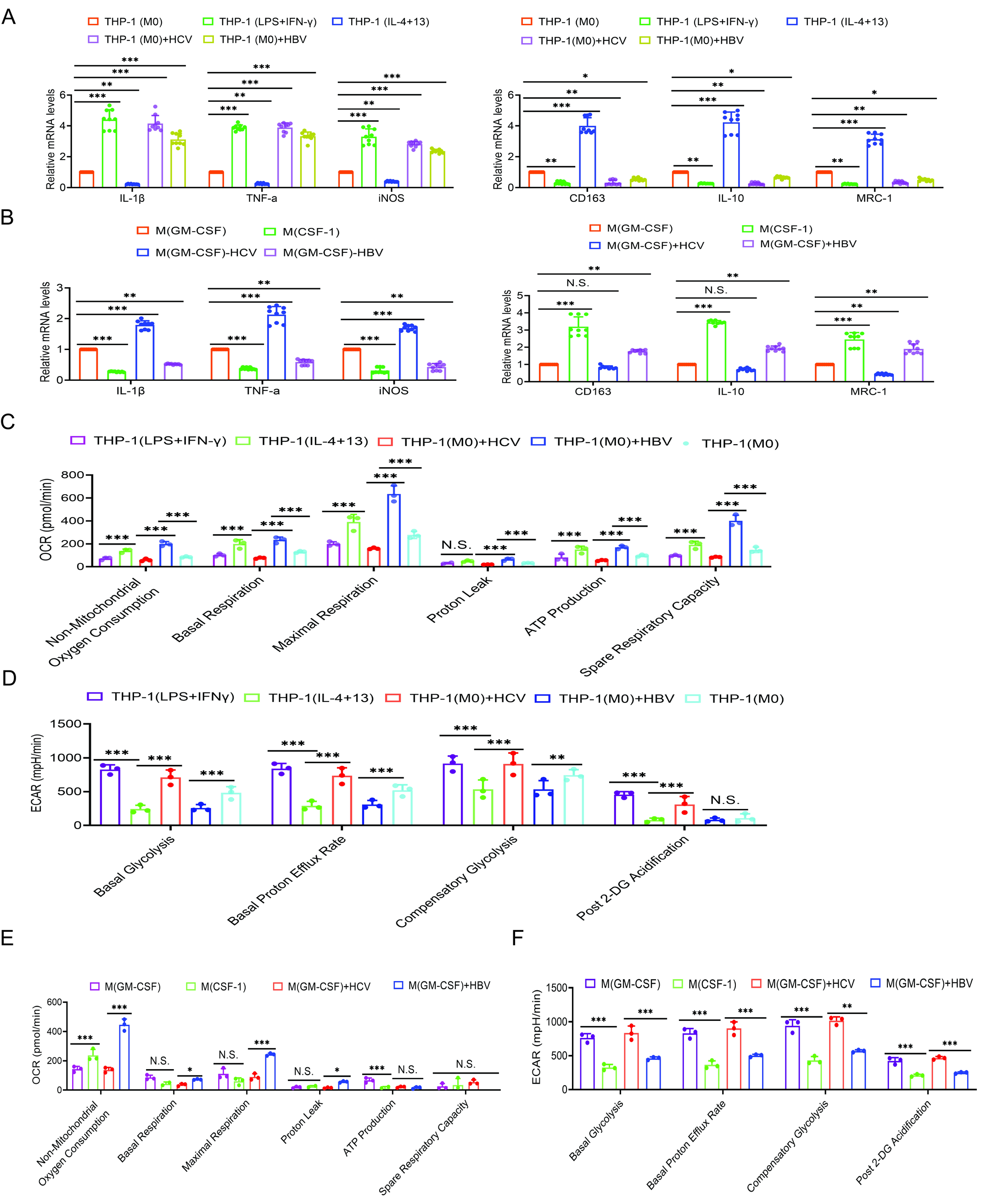

Supplement: S1 Fig — (A) THP-1 macrophages (THP-1(M0)) were treated with LPS and IFN-γ (THP-1(LPS+IFN-γ), with IL-4 and IL-13 (THP-1(IL-4+13)), with the incubation medium of Huh7 cells that had been infected with HCV (THP-1(M0)+HCV) for 24 hours, or with the incubation medium of Huh7 cells that had been transfected with the 1.3mer HBV genomic DNA (THP-1(M0)+HBV) for 48 hours. Cells were then lysed for the analysis of IL-1β, TNF-α, iNOS, CD163, IL-10 and MRC-1 RNAs by RT-qPCR. (B) Human MDMs were treated with GM-CSF (M(GM-CSF) or CSF-1 (M(CSF-1) for the induction of M1-like or M2-like polarization, respectively. In addition, M(GM-CSF) were also treated with HCV or HBV. Cells were then lysed for analysis of IL-1β, TNF-α, iNOS, CD163, IL-10 and MRC-1 RNAs by RT-qPCR. (C-D) and (E-F) are the quantitative analyses of the metabolic parameters of OCRs and ECARs of Fig 1A and 1B, respectively. N.S., not significant; *, p<0.05; **, p<0.01; ***, p<0.001. (TIF) [file ppat.1012079.s001.tif]

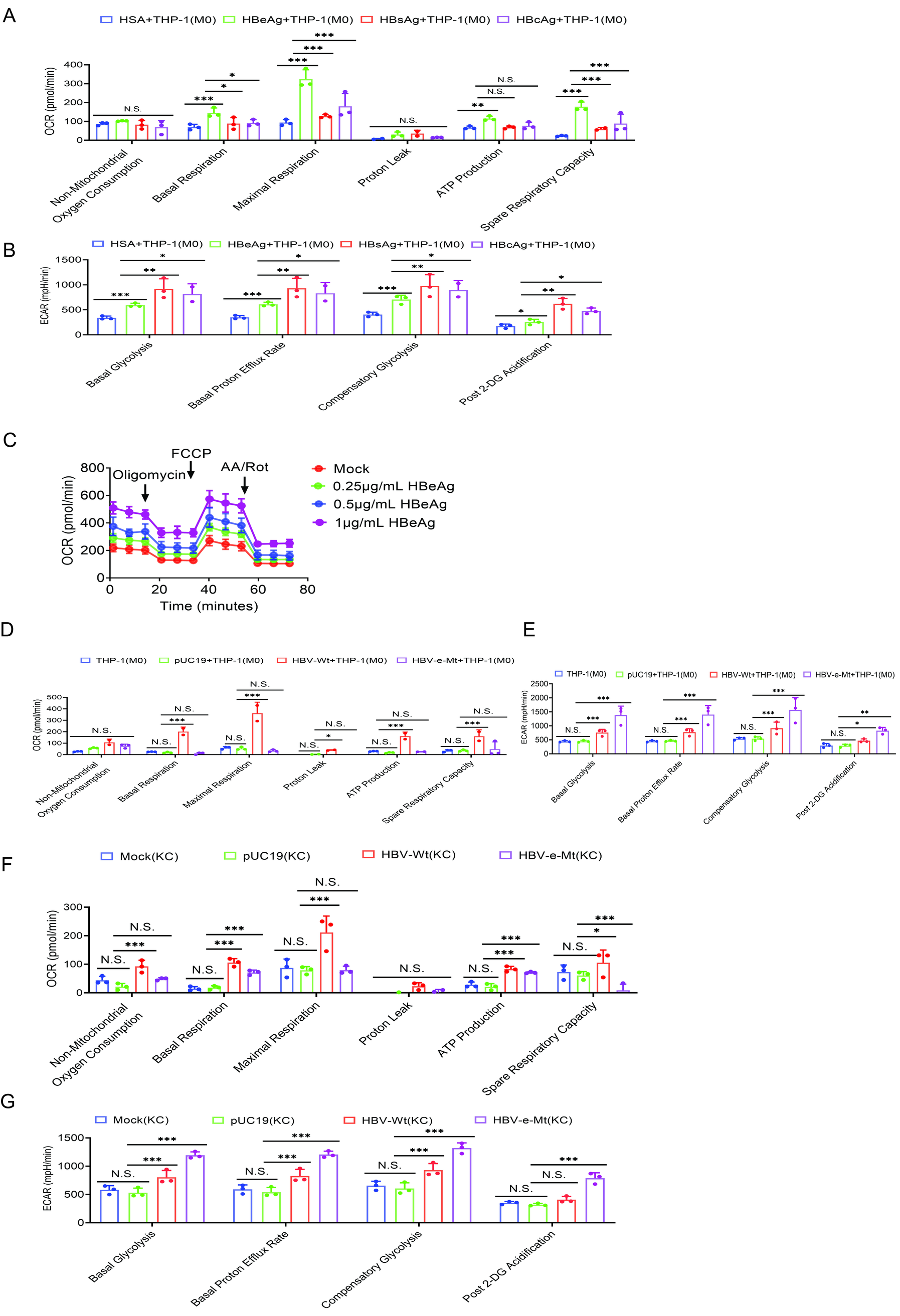

Supplement: S2 Fig — (A-B), (D-E) and (F-G) are the quantitative analyses of the metabolic parameters of OCRs and ECARs of Fig 2A–2C, respectively. (C) Analysis of the OCR of THP-1 macrophages that were treated with 0.25, 0.5 or 1 μg/mL of HBeAg for 48 hours. THP-1 macrophages not treated (Mock) was used as the control. N.S., not significant; *, p<0.05; **, p<0.01; ***, p<0.001. (TIF) [file ppat.1012079.s002.tif]

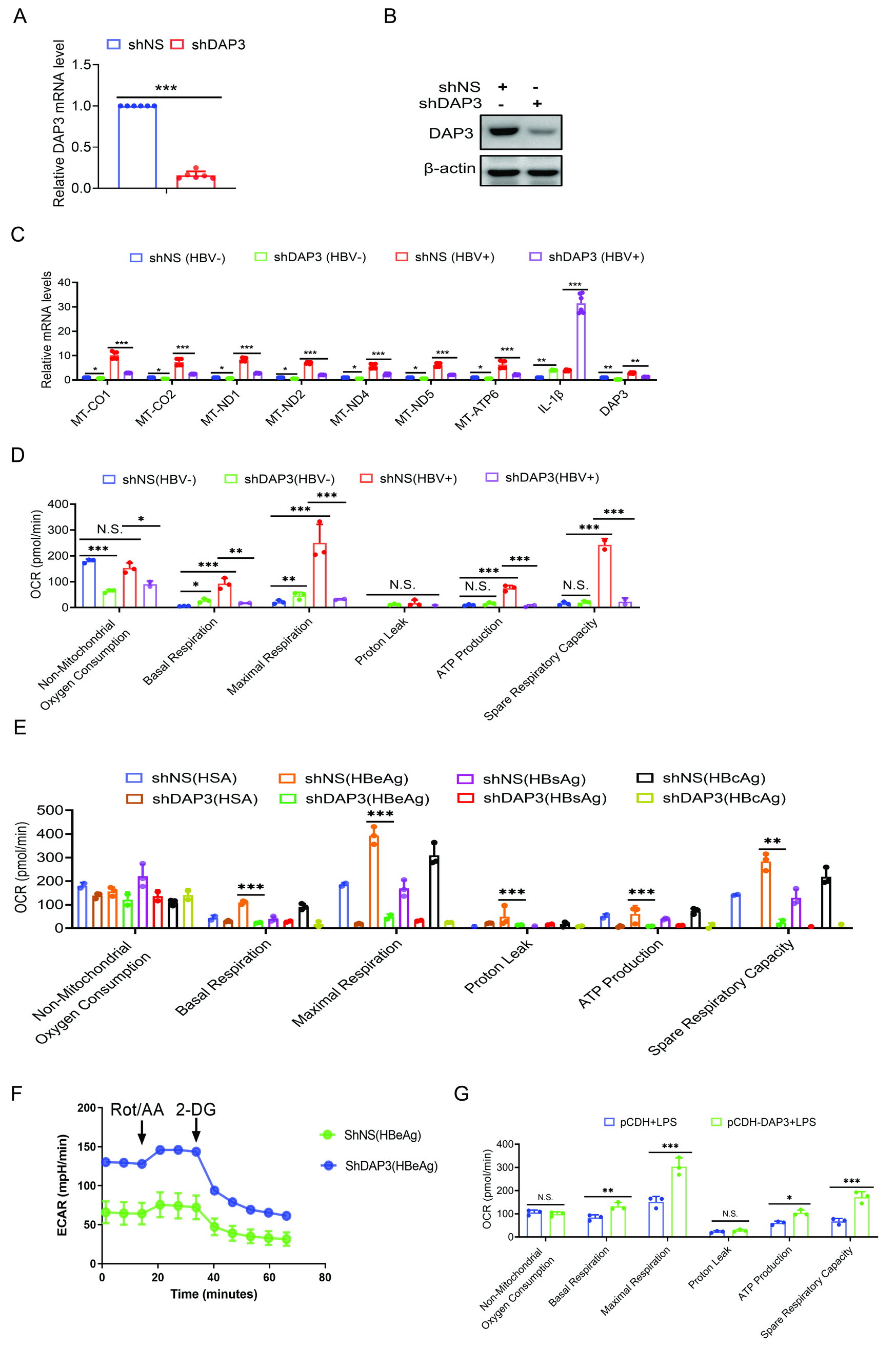

Supplement: S3 Fig — (A) RT-qPCR analysis of relative DAP3 RNA levels in THP-1 macrophages with (shDAP3) and without (shNS) DAP3 silencing. The level of DAP3 RNA without silencing was arbitrarily defined as 1. (B) Immunoblot analysis of DAP3 in THP-1 macrophages with and without DAP3 silencing. (C) The relative RNA levels of mitochondrial genes, IL-1β and DAP3 in THP-1 macrophages with and without DAP3 silencing and with and without HBV treatment were determined by RT-qPCR. (D), (E) and (G) are the metabolic parameters of the OCR of THP-1 macrophages shown in Fig 3F–3H, respectively. (F) THP-1 macrophages with and without DAP3 silencing were treated with 1 μg/mL HBeAg for 48 hours and then subjected to Seahorse analysis for ECAR. N.S., not significant; *, p<0.05; **, p<0.01; ***, p<0.001. (TIF) [file ppat.1012079.s003.tif]

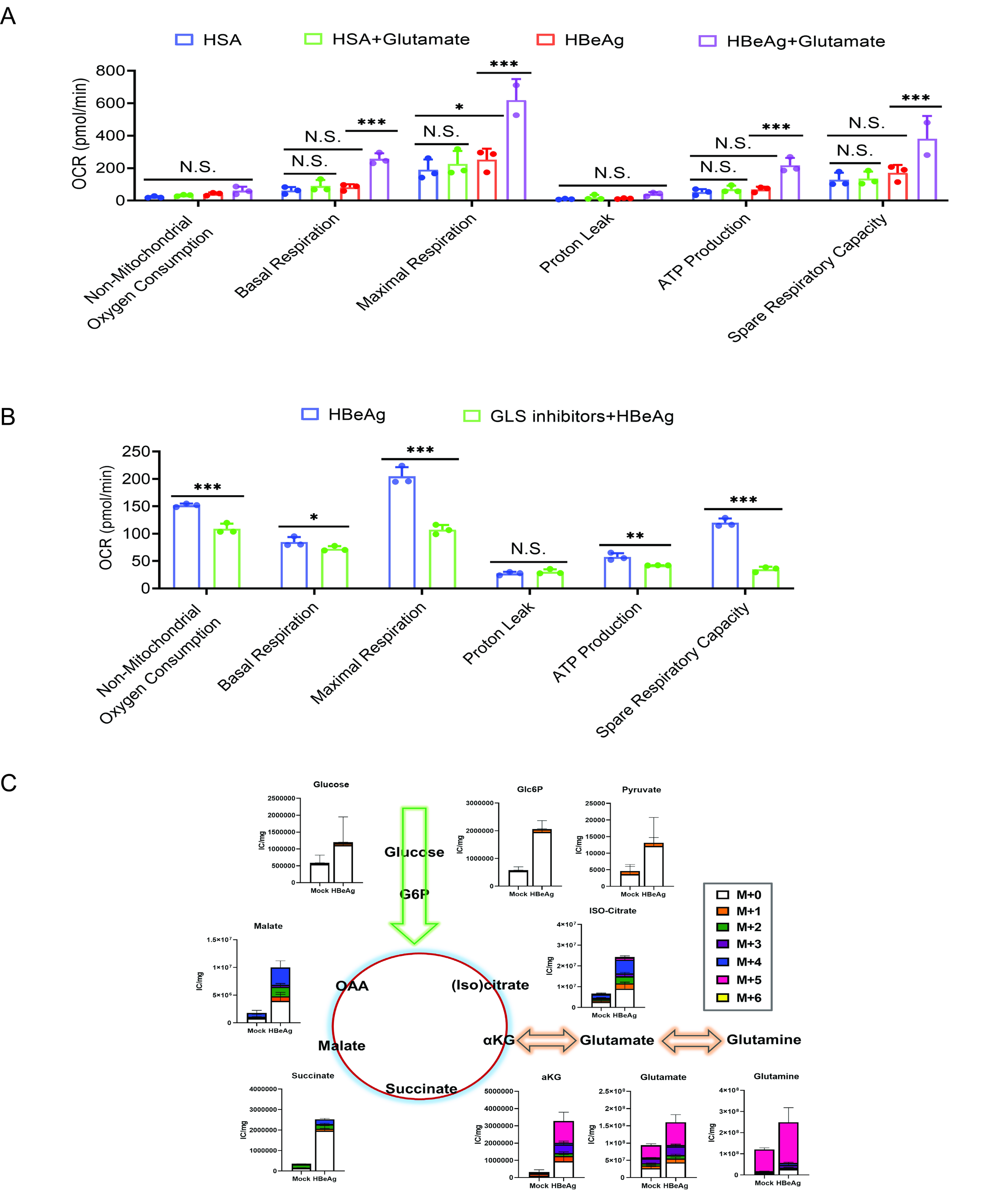

Supplement: S4 Fig — (A) and (B) are the quantitative analyses of individual metabolic parameters of the OCR studies shown in Fig 4G and 4H, respectively. N.S., not significant; *, p<0.05; **, p<0.01; ***, p<0.001. (C) The [U-13C]glutamine-tracing study on THP-1 macrophages with and without HBeAg treatment. (TIF) [file ppat.1012079.s004.tif]

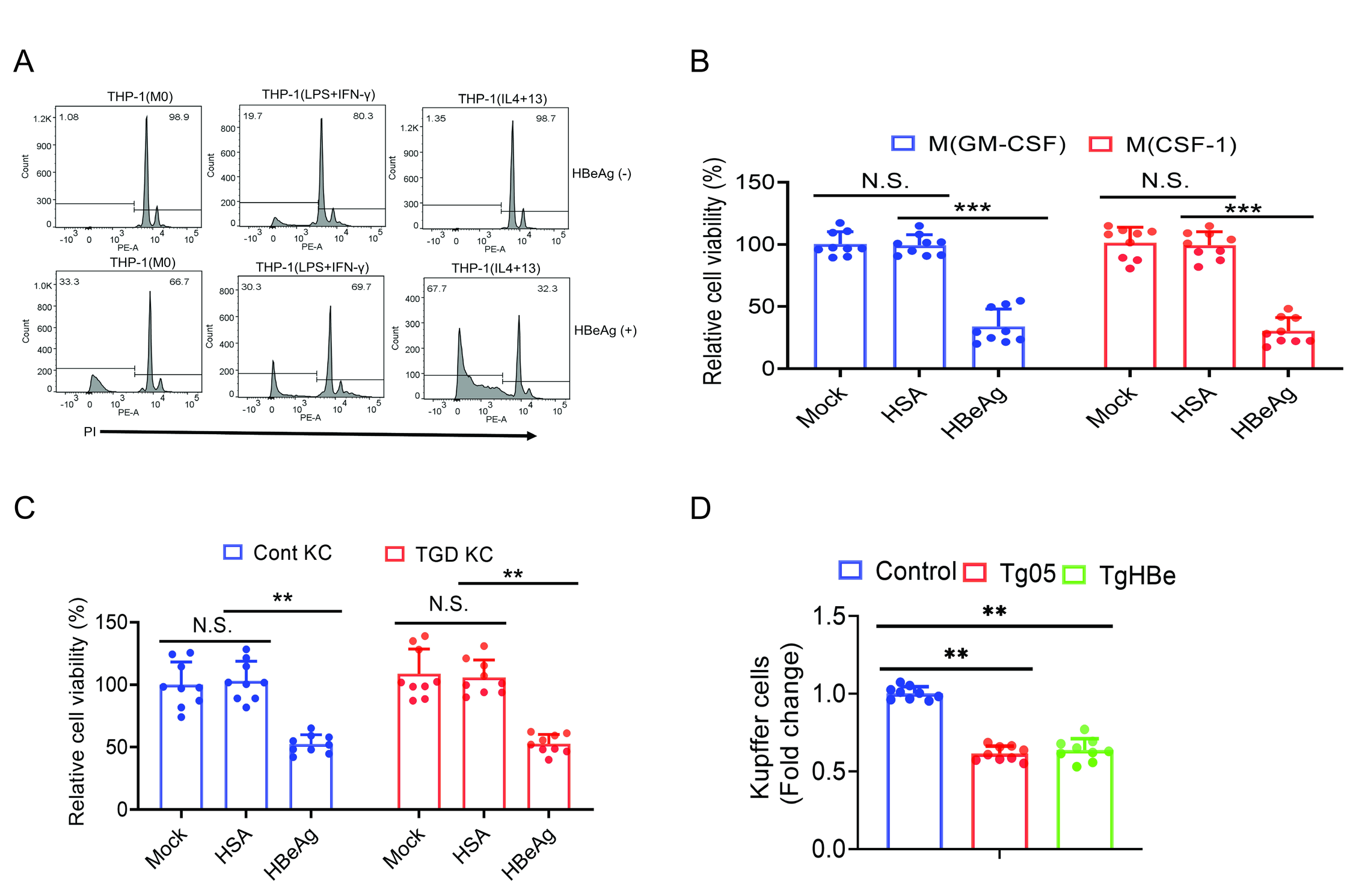

Supplement: S5 Fig — (A) THP-1 M0, M1-like and M2-like macrophages with and without the treatment of HBeAg were stained with propidium iodide and then analyzed by flow cytometry. (B) Human MDMs treated with GM-CSF for the induction of M1-like polarization or with CSF-1 for M2-like polarization were analyzed for their viability using the CCK8 assay. (C) Kupffer cells isolated from control mice (Cont KC) or HBV-negative mice born to hemizygous HBV transgenic dams (TGD KC) were analyzed for their viability using the CCK8 assay. (D) Kupffer cells were isolated from control mice and HBV transgenic mice carrying either the 1.3mer HBV genome (Tg05) or the precore protein gene (TgHBe) as described in the Methods section for analysis. Both Tg05 and TgHBe mice had reduced numbers of Kupffer cells. In (B), (C) and (D), N.S., not significant; **, p<0.01; ***, p<0.001. (TIF) [file ppat.1012079.s005.tif]

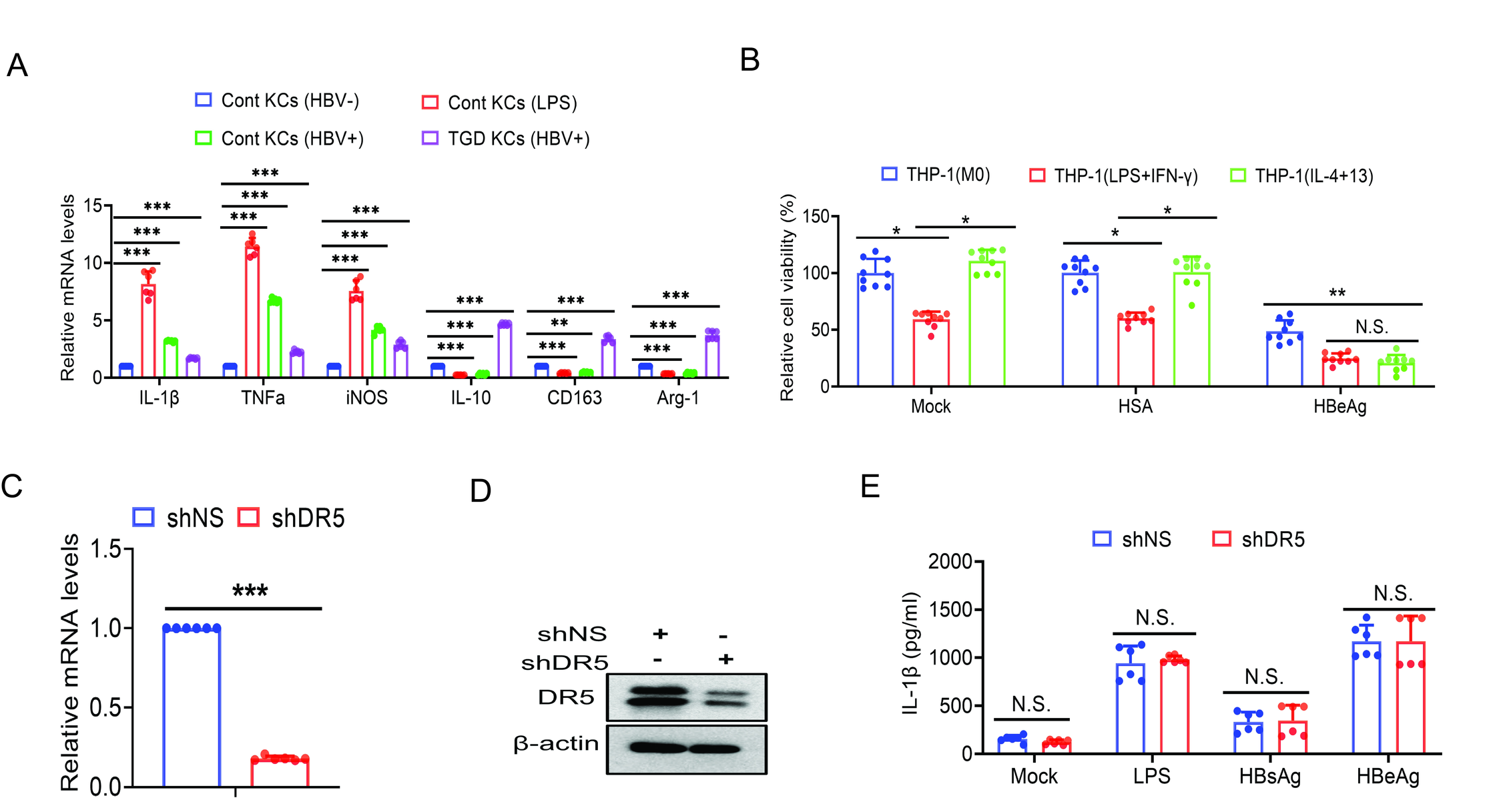

Supplement: S6 Fig — (A) Kupffer cells isolated from control mice or TGD mice that had been injected with pUC19 (HBV-) or the 1.3mer HBV genomic DNA (HBV+) were analyzed for the RNA levels of M1 markers (i.e., IL-1β and TNF-α) and M2 markers (i.e., IL-10 and CD163) by RT-qPCR. Control mice intraperitoneally injected with LPS (1 mg/kg) for 48 hours were also analyzed to serve as the control. (B) THP-1 macrophages without (M0) or with induction of M1-like polarization (THP-1(LPS+IFN-γ)) or M2-like polarization (THP-1(IL4+13)) and without (mock) or with the treatment of HSA or HBeAg were analyzed for their viability using the CCK8 assay. (C) THP-1 macrophages without (shNS) or with DR5 silencing (shDR5) were lysed for the analysis of DR5 RNA by RT-qPCR. (D) THP-1 macrophages as described in (C) were lysed for immunoblot analysis of DR5. (E) The incubation media of THP-1 macrophages without (shNS) or with DR5 silencing (shDR5) and without (mock) or with the treatment of LPS, HBsAg or HBeAg were collected and analyzed for the level of IL-1β using ELISA. In (A-C) and (E), N.S., not significant; *, p<0.05; **, p<0.01; ***, p<0.001. (TIF) [file ppat.1012079.s006.tif]

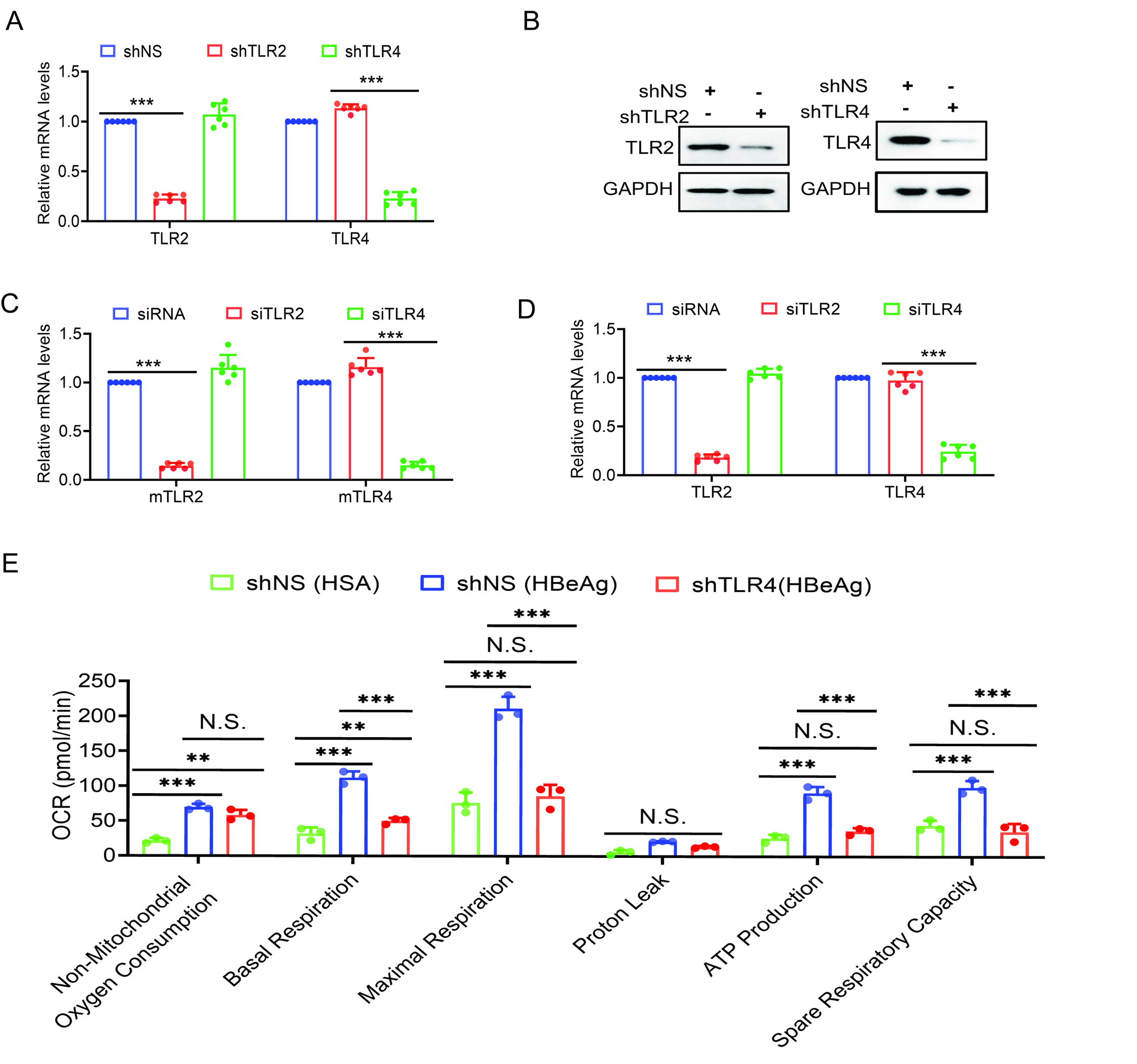

Supplement: S7 Fig — (A) THP-1 macrophages with shNS, shTLR2 or shTLR4 silencing were analyzed for the RNA level of TLR2 and TLR4 by RT-qPCR. (B) Immunoblot analysis of THP-1 macrophages with shNS, ShTLR2 or shTLR4 silencing. (C) Kupffer cells treated with control siRNA (siNS), TLR2 siRNA (siTLR2) or TLR4 siRNA (siTLR4) were lysed for analysis of mouse TLR2 (mTLR2) and TLR4 (mTLR4) RNAs by RT-qPCR. (D) Human MDMs treated with siNS, siTLR2 or siTLR4 were lysed for RNA analysis by RT-qPCR. (E) Quantitative analysis of the metabolic parameters of the OCR study shown in Fig 7H. In (A) and (C-E), N.S., not significant; *, p<0.05; **, p<0.01; ***, p<0.001. (TIF) [file ppat.1012079.s007.tif]
