# Supplementary material for: Hepatitis B virus e antigen induces atypical metabolism and differentially regulates programmed cell deaths of macrophages
Source: PLoS Pathog. 2024 Mar 11;20(3):e1012079. doi: 10.1371/journal.ppat.1012079 (PMC10957081; doi:10.1371/journal.ppat.1012079)
Supplement: S2 Data — (PDF) [file ppat.1012079.s009.pdf]

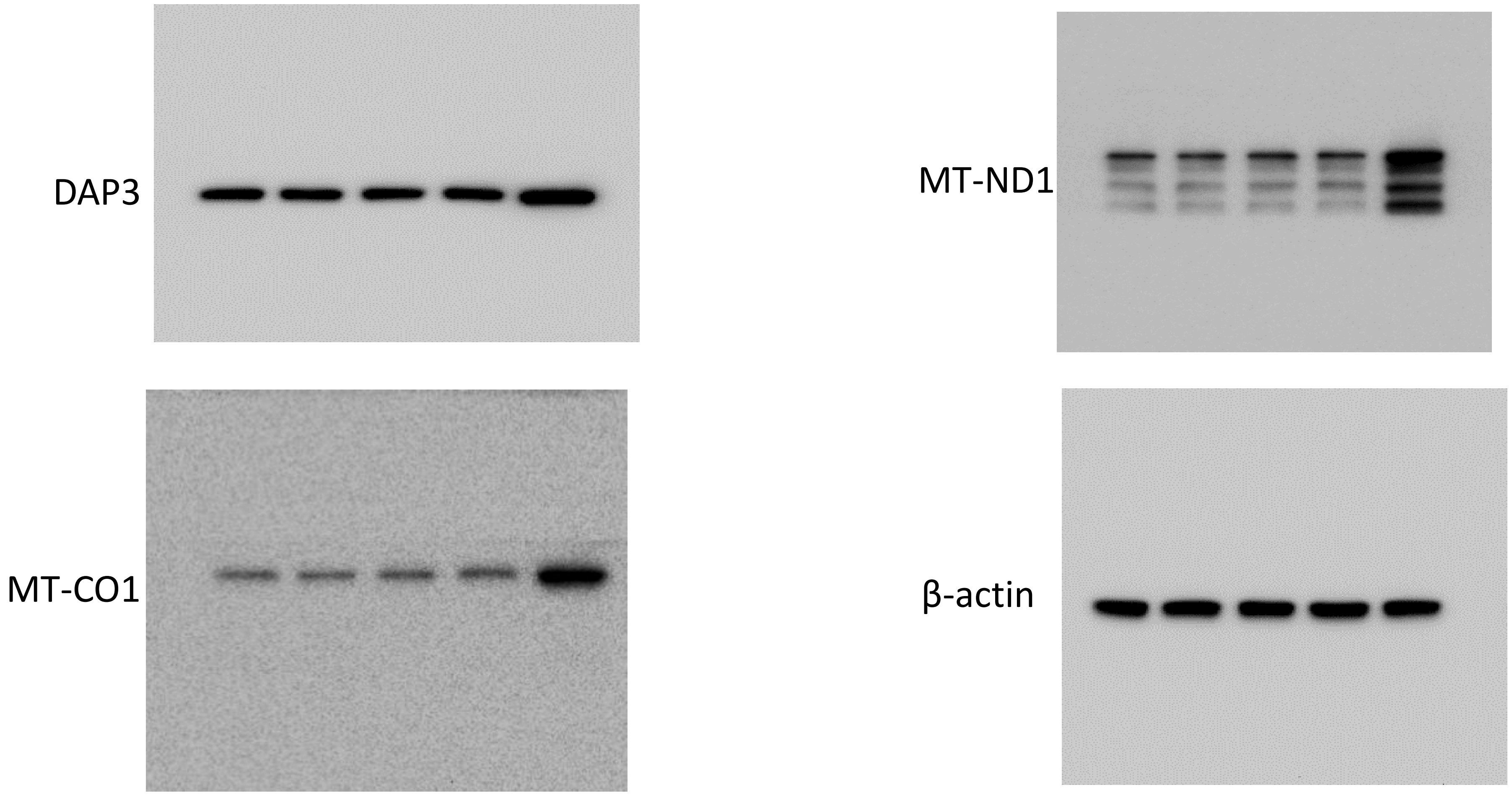

Unedited gel for Fig 3C

DAP3

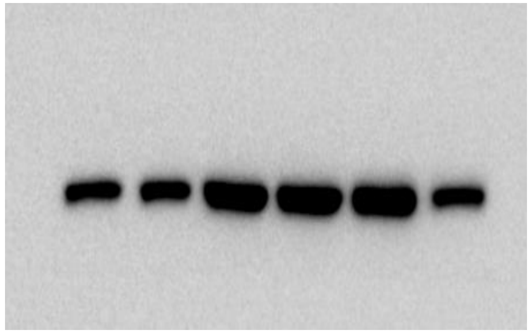

MT-ND1

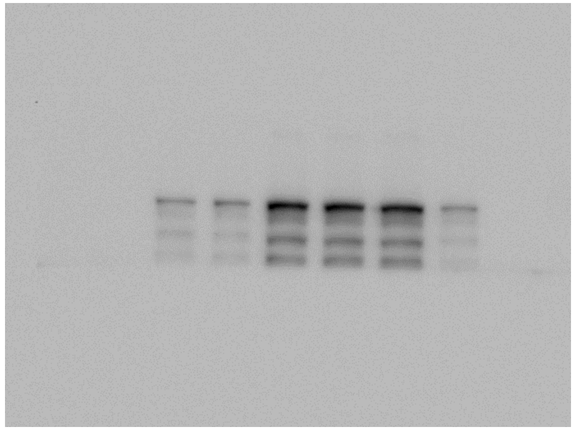

MT-CO1

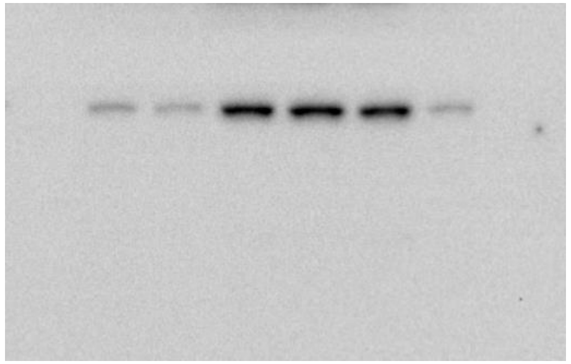

β-actin

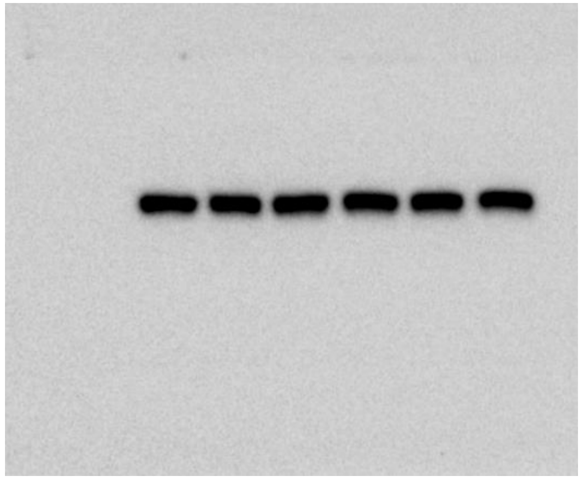

Unedited gel for Fig 3E

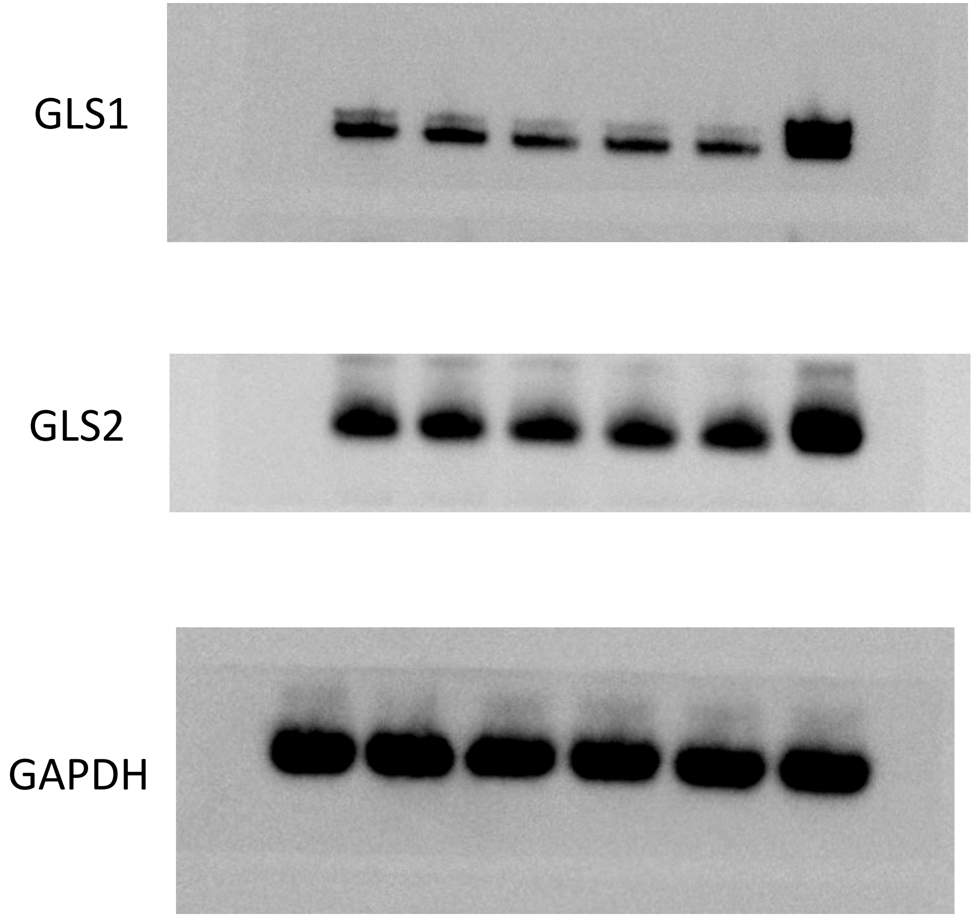

Unedited gel for Fig 4E

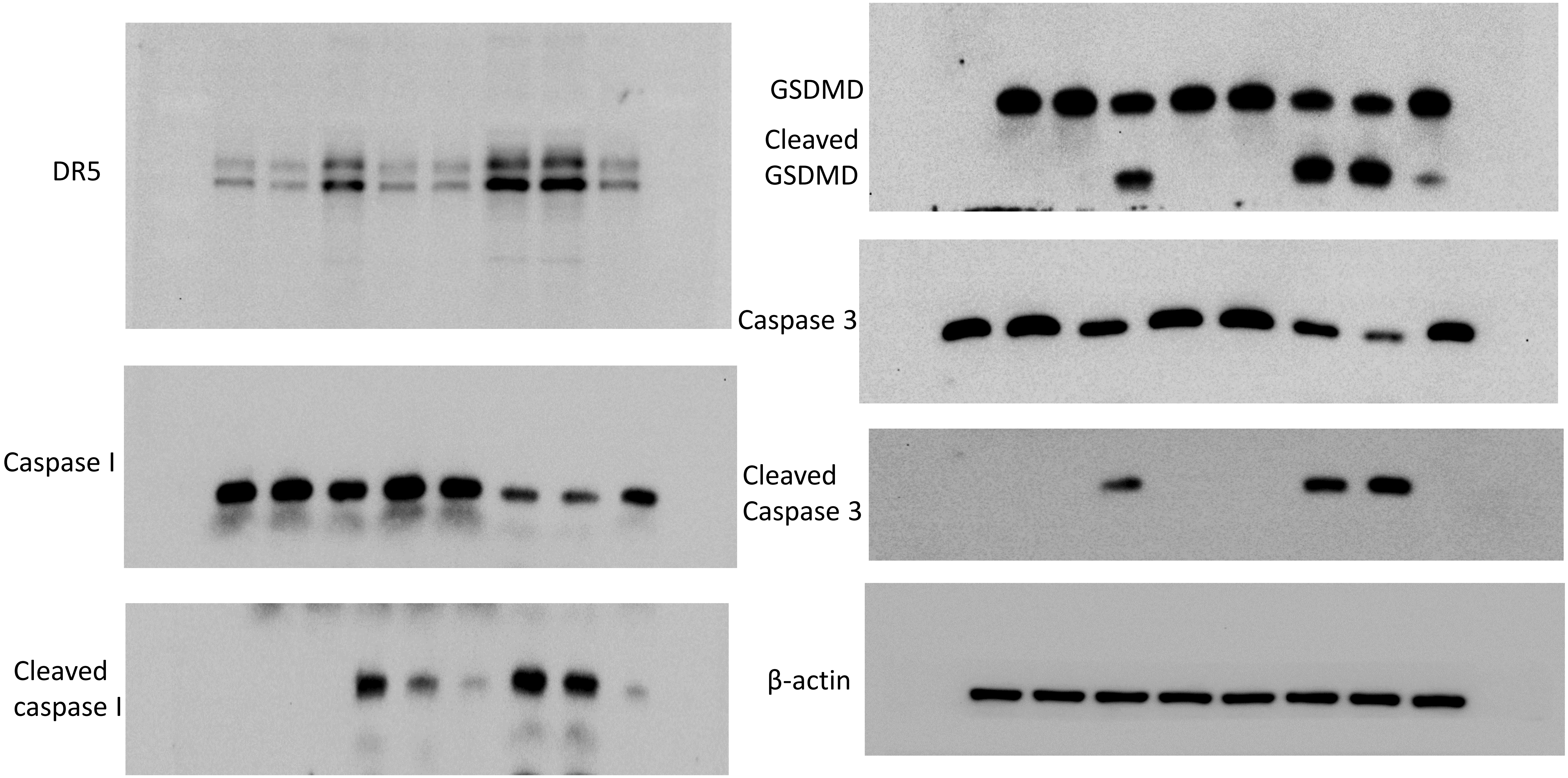

Unedited gel for Fig 6C

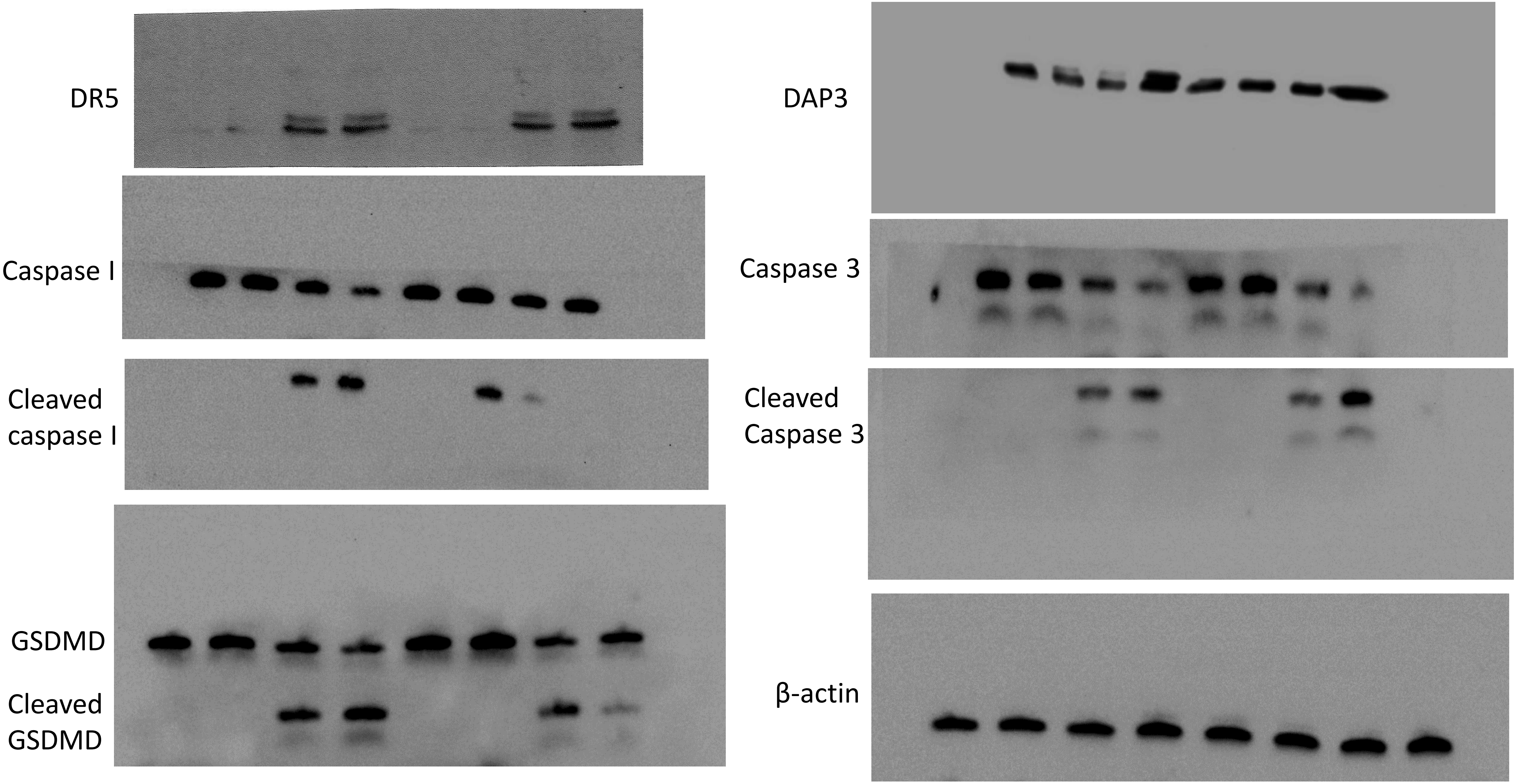

Unedited gel for Fig 6D

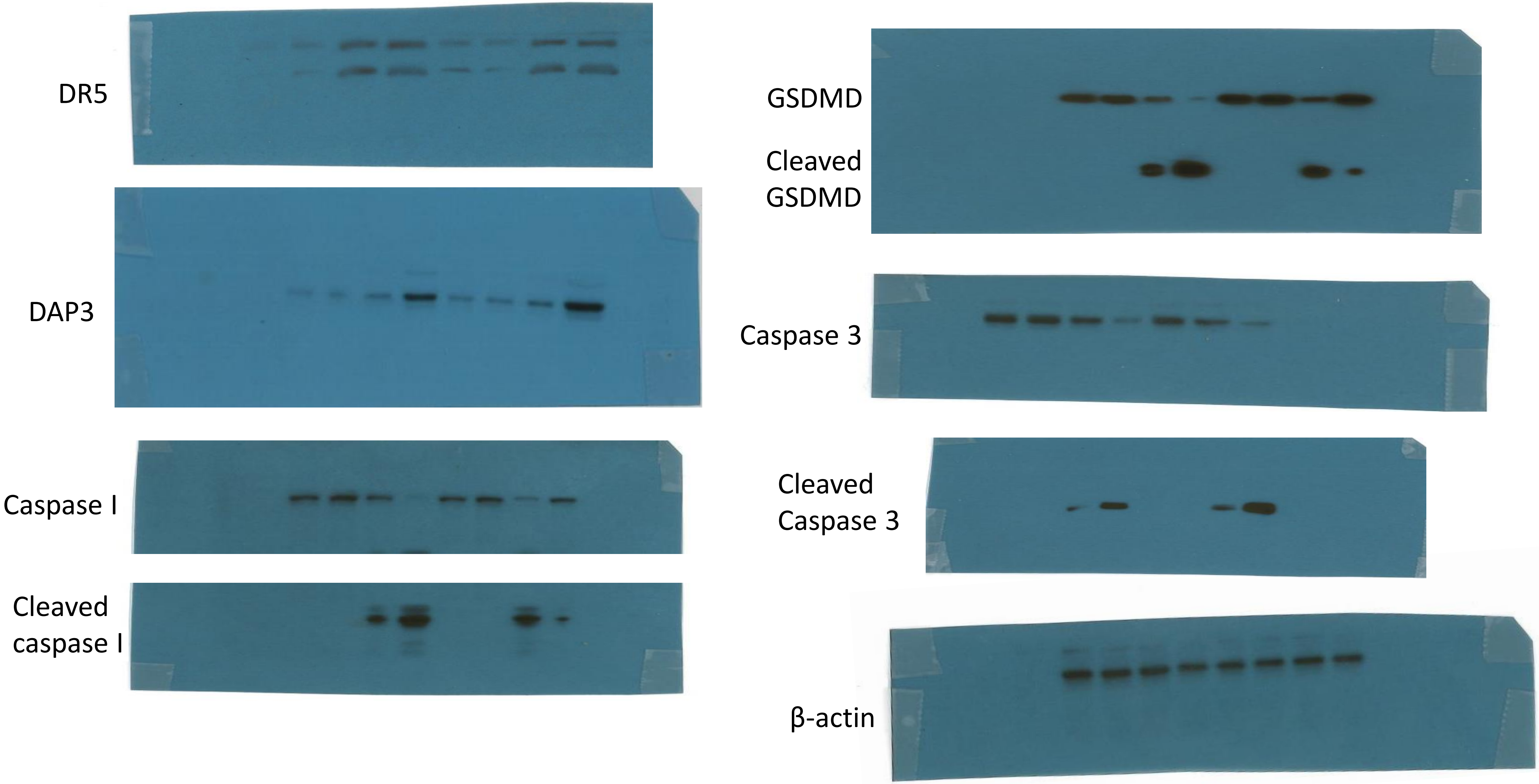

Unedited gel for Fig 6E

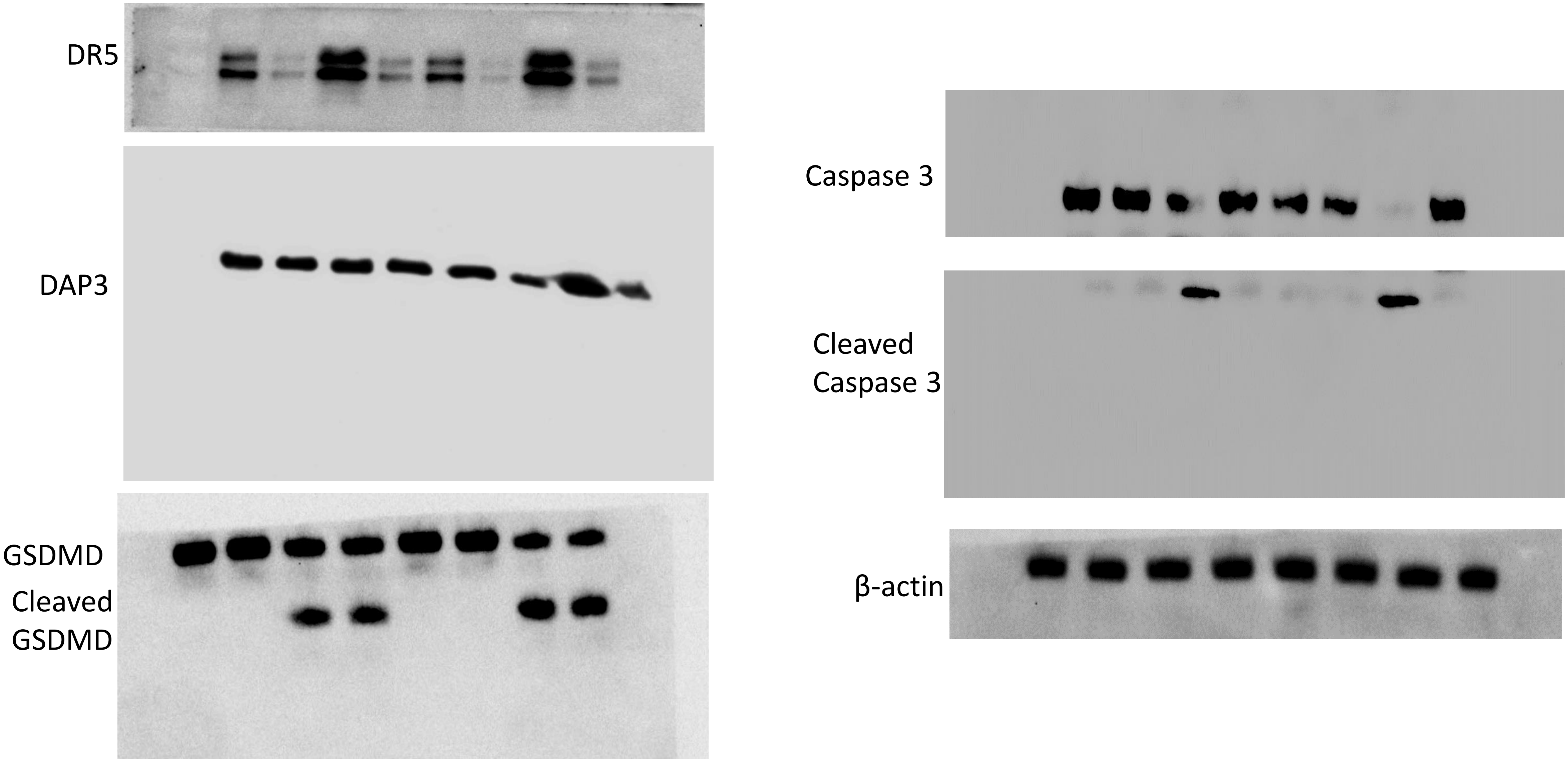

Unedited gel for Fig 6F

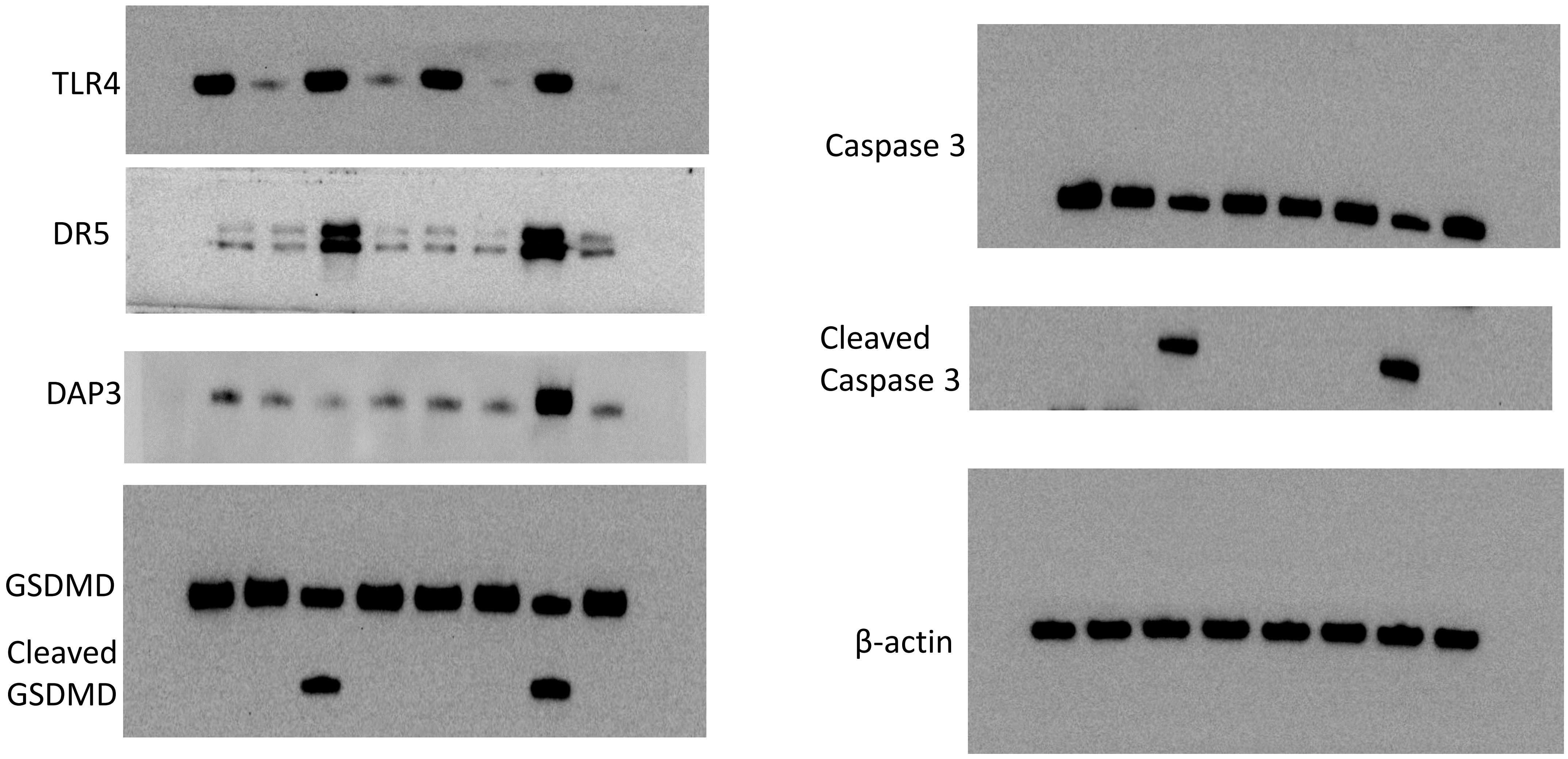

Unedited gel for Fig 7F

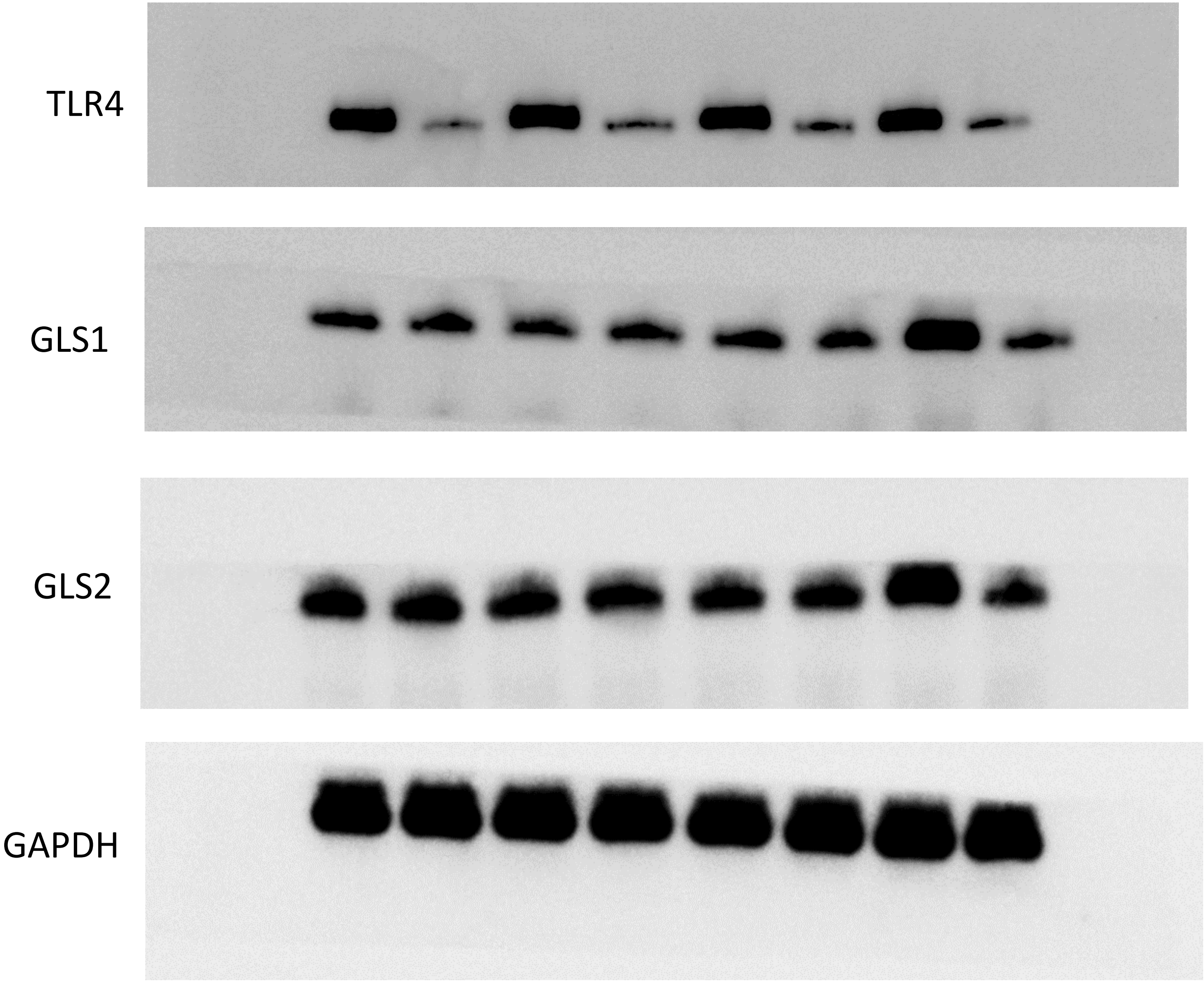

Unedited gel for Fig 7G

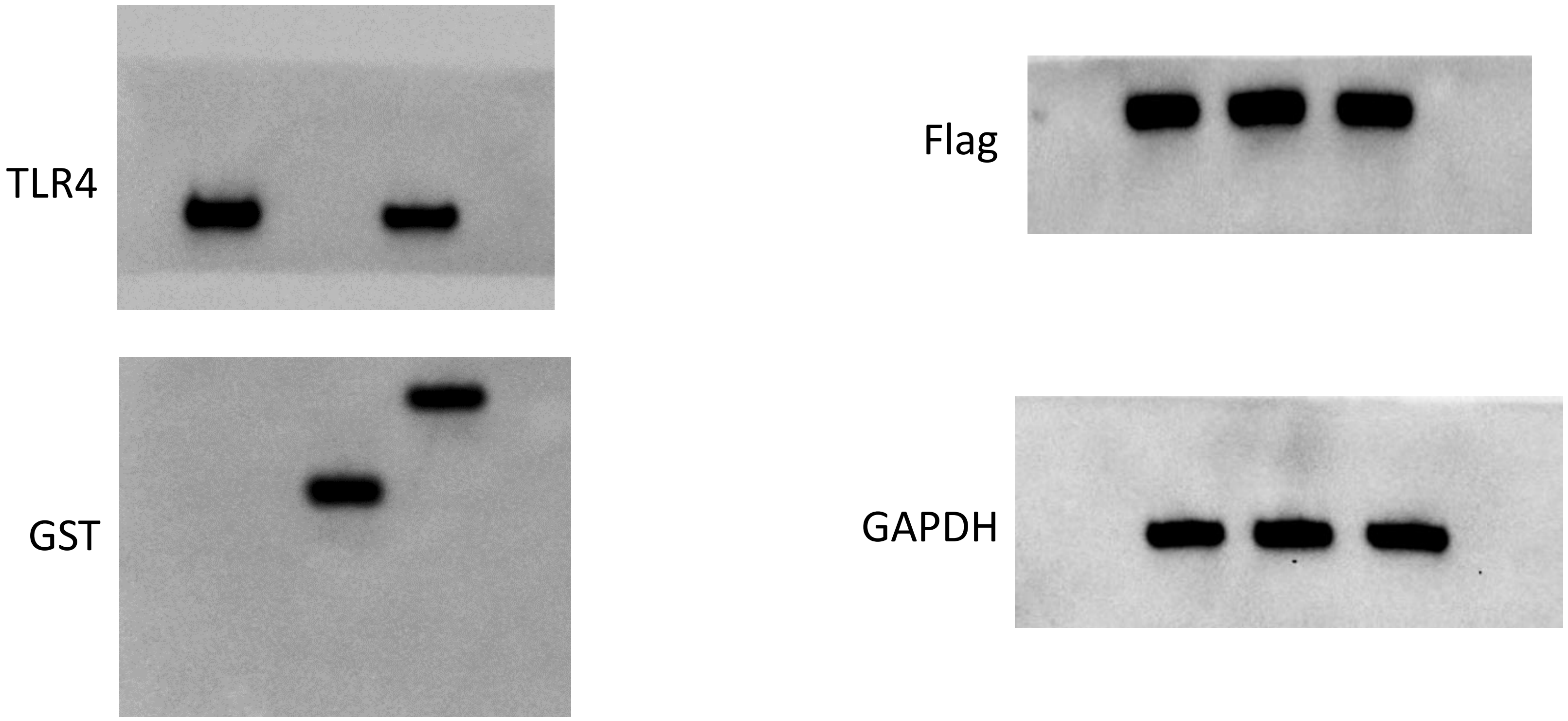

Unedited gel for Fig 7I

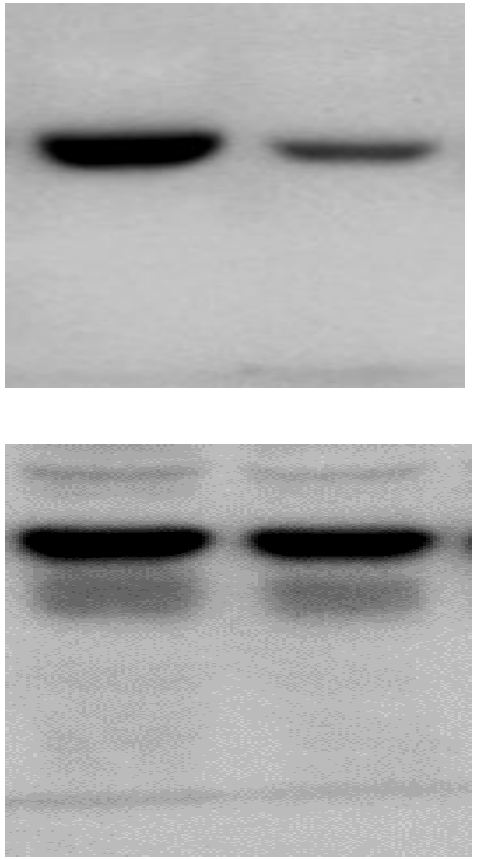

Unedited gel for S3B_Fig

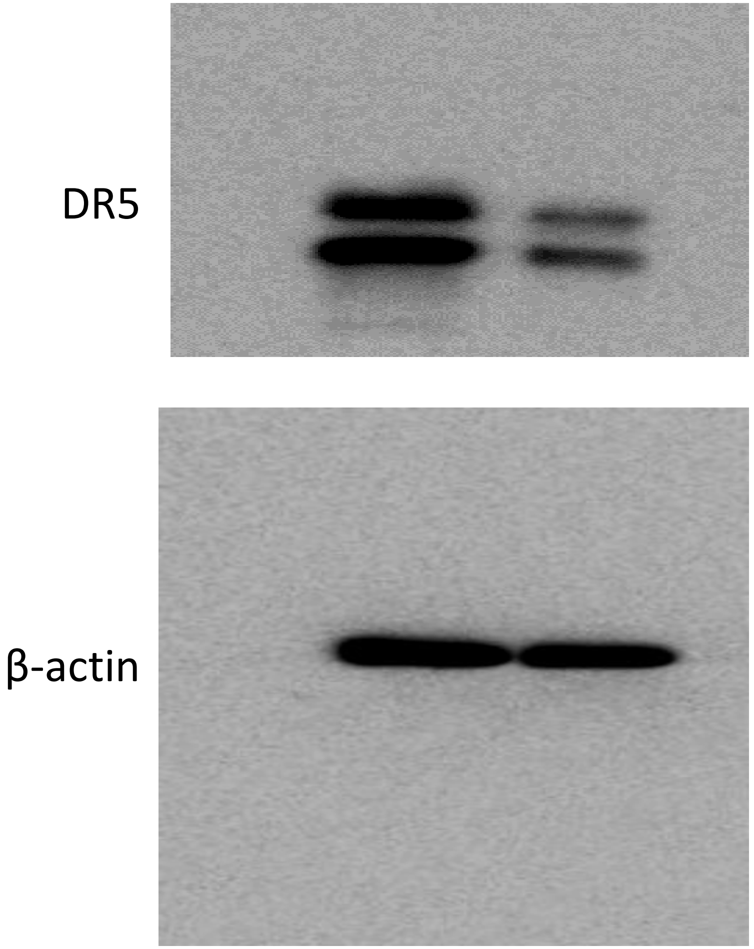

Unedited gel for S6D_Fig

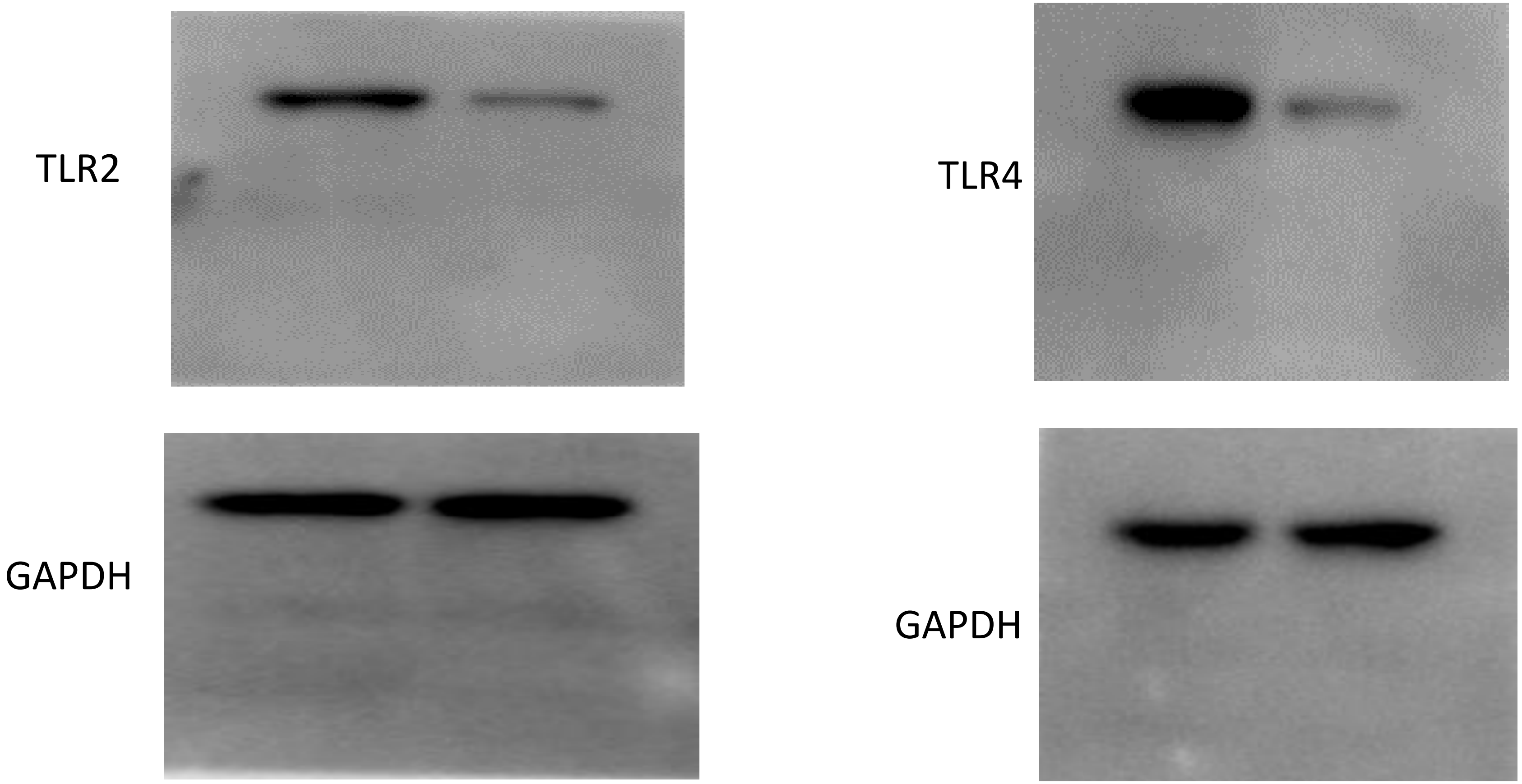

Unedited gel for S7B_Fig
